# Hormonal and proteomic analyses of southern blight disease caused by *Athelia rolfsii* and root chitosan priming on *Cannabis sativa* in an in vitro hydroponic system

**DOI:** 10.1002/pld3.528

**Published:** 2023-09-08

**Authors:** Pipob Suwanchaikasem, Shuai Nie, Jamie Selby‐Pham, Robert Walker, Berin A. Boughton, Alexander Idnurm

**Affiliations:** ^1^ School of BioSciences University of Melbourne Melbourne Victoria Australia; ^2^ Mass Spectrometry and Proteomics Facility, Bio21 Molecular Science and Biotechnology Institute University of Melbourne Melbourne Victoria Australia; ^3^ Cannabis and Biostimulants Research Group Pty Ltd Melbourne Victoria Australia; ^4^ Australian National Phenome Centre Murdoch University Perth Western Australia Australia

**Keywords:** cell wall‐degrading enzymes, chitinase, exudate, peroxidase, plant defense, proteomics

## Abstract

Southern blight disease, caused by the fungal pathogen *Athelia rolfsii*, suppresses plant growth and reduces product yield in *Cannabis sativa* agriculture. Mechanisms of pathology of this soil‐borne disease remain poorly understood, with disease management strategies reliant upon broad‐spectrum antifungal use. Exposure to chitosan, a natural elicitor, has been proposed as an alternative method to control diverse fungal diseases in an eco‐friendly manner. In this study, *C. sativa* plants were grown in the Root‐TRAPR system, a transparent hydroponic growth device, where plant roots were primed with .2% colloidal chitosan prior to *A. rolfsii* inoculation. Both chitosan‐primed and unprimed inoculated plants displayed classical symptoms of wilting and yellowish leaves, indicating successful infection. Non‐primed infected plants showed increased shoot defense responses with doubling of peroxidase and chitinase activities. The levels of growth and defense hormones including auxin, cytokinin, and jasmonic acid were increased 2–5‐fold. In chitosan‐primed infected plants, shoot peroxidase activity and phytohormone levels were decreased 1.5–4‐fold relative to the unprimed infected plants. When compared with shoots, roots were less impacted by *A. rolfsii* infection, but the pathogen secreted cell wall‐degrading enzymes into the root‐growth solution. Chitosan priming inhibited root growth, with root lengths of chitosan‐primed plants approximately 65% shorter than the control, but activated root defense responses, with root peroxidase activity increased 2.7‐fold along with increased secretion of defense proteins. The results suggest that chitosan could be an alternative platform to manage southern blight disease in *C. sativa* cultivation; however, further optimization is required to maximize effectiveness of chitosan.

## 1 INTRODUCTION

*Cannabis sativa* is a versatile crop, offering a range of benefits to civilization for millennia. Two *C. sativa* varieties, industrial hemp and medicinal cannabis, are similar in terms of botanical characterization but differ in application and usage of tissues (Chandra et al., 2017). Seeds of industrial hemp are used as food supplements, while fibers are used for making cloth, textile, paper, animal bedding, composite materials, and building blocks. Leaves and flowering heads of medicinal cannabis contain psychoactive secondary metabolites, cannabinoids, which are used for treating neurological disorders such as epilepsy, anxiety, depression, and neuropathic pain (Chandra et al., 2017). The combined global markets of industrial hemp and medicinal cannabis were valued $17.8 billion USD in 2021. The market size is projected to reach $134.4 billion USD by 2030, exceeding the market sizes of some horticultural crops such as peanut, strawberry, and avocado (Grand View Research, 2022).

However, growing *C. sativa* plants is challenging and can be problematic. For example, cultivating *C. sativa* needs approval and compliance due to the controlled nature of the plant's secondary metabolites (Bodwitch et al., 2021). Improper environmental conditions such as compacted soil, insufficient nutrients, high temperature, low light, and poor irrigation suppress plant growth and reduce product yield (García‐Tejero et al., 2019; Tang et al., 2016). *C. sativa* is also susceptible to biotic stresses, with various pests and pathogens having been recorded (McPartland et al., 2000; Wang, 2021). In terms of fungal diseases, *Botrytis cinerea* causing gray mold and bud rot and *Pythium* and *Fusarium* species causing crown and root rot have been reported in Canada (Punja, 2021).

In Australia, *Athelia rolfsii* (stem and root rot), *Macrophomina phaseolina* (charcoal root rot), *Fusarium* sp. (crown lesion), and *Phomopsis* sp. (stem canker) have been isolated from diseased *C. sativa* (Department of Agriculture and Fisheries Queensland Government, n.d.) but have not been further characterized. *A. rolfsii* causing southern blight disease has been increasingly identified to impact *C. sativa* plantations worldwide. Recently, it was isolated from industrial hemp in Virginia and Louisiana, USA, and Crete, Greece (Amaradasa et al., 2020; Chatzaki et al., 2022; Singh et al., 2022). Infected plants show wilting symptoms, with yellow wilted leaves, and a lower brown stem alongside appearances of cottony white mycelia and dark brown sclerotia near the crown. To date, research on *C. sativa* pathogens has been limited, and disease management programs have not been well established (Punja, 2021; Sandler et al., 2019).

In crop production, fungal diseases are estimated to cause approximately 11% to 13% annual loss in the 21st century (Moore et al., 2021). To control disease, synthetic fungicides have been widely applied, but crop loss has not been significantly improved in the past 40 years (Moore et al., 2021), alongside the potential negative impacts of fungicides in the environment. For example, azoxystrobin at commercial doses (ranging from .001 to .1 ppm) was detected to impair developmental processes and antioxidant enzymatic activities of zebrafish embryos in laboratory conditions (Vieira et al., 2021). In a field experiment, the soil microbial community was significantly modified after 14 and 35 days of tebuconazole application at the recommended agronomic dose (Storck et al., 2018). Moreover, in medicinal cannabis production, the uses of fungicides are highly restricted, and the plant products must be tested for fungicide residues where strict standards are applied (Craven et al., 2019). With increasing concerns in human health and environmental safety, natural products could be an alternative platform for fungal disease management.

Chitosan is one such natural product, with its protective effect demonstrated in various plants against different diseases (Malerba & Cerana, 2016; Riseh et al., 2022). Regarding southern blight disease, priming carrot seeds (*Daucus carota*) with 1% chitosan for 12 h before sowing reduced incidence of the disease on carrot roots by 30% to 42% (Rahman et al., 2021). Treating chili seeds (*Capsicum annuum*) with alginate beads, containing plant growth‐promoting bacteria, *Bacillus licheniformis*, and chitosan nanoparticles (25 mg mL^−1^) diminished southern blight disease occurrence by 33% (Panichikkal et al., 2021). Spraying cowpea (*Vigna unguiculata*) first leaves with 2–6 mg mL^−1^ chitosan also reduced symptoms of southern blight by 27% to 60% (de Souza et al., 2018). The mechanism underlying chitosan eliciting properties is correlated to the activation of secondary messengers such as hydrogen peroxide (H_2_O_2_) and nitric oxide (NO) and defense hormones such as jasmonic acid (JA) and salicylic acid (SA), resulting in enhanced productions of defense metabolites such as phytoalexins and phenolic compounds and defense‐related enzymes such as peroxidase, catalase, and chitinase (Pichyangkura & Chadchawan, 2015). Chitosan can also induce callose and lignin deposition to the plant cell wall, strengthening the plant's physical barriers against fungal invasion (De Vega et al., 2021; Kuyyogsuy et al., 2018). However, further exploration is required to advance understanding in this area. Characterization of plant receptors that can specifically bind chitosan and the downstream signaling molecules that are triggered upon chitosan induction will clarify the effects of chitosan on plant defense and could promote the uses of chitosan. In addition, previous studies have predominantly conducted chitosan treatments on foliar tissues, pre‐germinated seeds or postharvest fruits (Lopez‐Moya et al., 2019). Root treatment including soil amendment and irrigation or in hydroponic solution has been less investigated but could be a convenient and effective platform to manage fungal diseases, especially against soil‐borne pathogens like *A. rolfsii*.

The eliciting effect of chitosan on the *C. sativa* root defense systems was recently reported. These results showed that .2% to .5% colloidal chitosan can induce production and secretion of defense proteins in root tissues and exudates (Suwanchaikasem et al., 2023). This was the impetus for further study to examine whether chitosan‐primed plants will be more resistant against fungal diseases. Therefore, in this study, we explored the pathological effect of the *A. rolfsii* fungus on *C. sativa* and the root priming effect of chitosan to enhance whole‐plant defense responses against the infection, monitoring plant morphology, growth parameters, phytohormone contents, defense enzyme activities, and exudate proteome profiles.

## 2 MATERIALS AND METHODS

### 2.1 Plant and fungal materials

*C. sativa* var. Ferimon seeds were kindly provided by the Southern Hemp Co., Australia. It is an industrial hemp seed‐type variety with less than .12% tetrahydrocannabinol (THC) content (IHempFarms, n.d.). Fungal pathogen *A. rolfsii* strain BRIP 39302a was obtained from the Plant Pathology Herbarium, Biological Collections, Department of Agriculture and Fisheries, Queensland Government, Australia. It was isolated from industrial hemp in Burnett Valley, Murgon, Queensland, in 2003 and identified by a plant pathologist, Dr Roger Shivas. The other pathogens isolated from *C. sativa* were *M. phaseolina* strain BRIP 39354a, *Fusarium* sp. strain BRIP 69154a, and *Phomopsis* sp. strain BRIP 70500a (Department of Agriculture and Fisheries Queensland Government, n.d.).

### 2.2 Chemicals

Medium molecular weight chitosan used in this study was 75% to 85% deacetylated (product number 448877, Sigma‐Aldrich, USA). Colloidal chitosan and Hoagland solution were prepared as previously described (Suwanchaikasem et al., 2022). Potato dextrose agar (PDA, product number CM0139) was sourced from Oxoid Limited, UK. Yeast nitrogen base (YNB, product number Y0626) and agar (product number A1296) were obtained from Sigma‐Aldrich, USA. Liquid chromatography–mass spectrometry (LC–MS) grade acetonitrile (ACN) and methanol were used for all biochemical analyses (Fisher Scientific, USA).

### 2.3 Antifungal effect of chitosan

Colloidal chitosan together with broad‐spectrum fungicides, tebuconazole, and azoxystrobin (Syngenta, Australia) were screened in vitro for antifungal effect against *A. rolfsii*. Chitosan was dispersed in a PDA into final concentrations of .2% and .5% w/v. Tebuconazole and azoxystrobin powder were dissolved in ACN and diluted thousand times in PDA to a final concentration of 10 μg mL^−1^. The mixture was poured into a small Petri dish (6 cm in diameter) and once it was set, a 5 × 5 mm disc of *A. rolfsii* mycelia was added to the center of the media. The diameter of fungal growth was recorded 3 days after culturing. Three biological replicates were performed per condition.

### 2.4 Testing chitosan consumption by *A. rolfsii*

Two concentrations of colloidal chitosan and glucose (.2% and .5% w/v) were dissolved in a YNB without amino acids and mixed with 1% agar. The media was set in a small Petri dish and a 5 mm disc of *A. rolfsii* mycelia was then added to the center. After incubating at room temperature for 7 days, fungal growth was monitored. Three biological replicates were performed per condition.

### 2.5 Plant‐pathogen experiment

#### 2.5.1 Seed germination and initiation (Day 0)

*C. sativa* var. Ferimon seeds were surface sterilized with 70% ethanol for 1 min and .04% sodium hypochlorite for 10 min and imbibed overnight. Seeds were germinated in a Petri dish with a moist filter paper for 3 days. Seeding with 4‐ to 6‐mm‐long taproot was transferred to the Root‐TRAPR system, supplied with a full‐strength Hoagland solution (*Day 0*). Seedlings were grown in a CMP 6010 growth chamber (Conviron, Canada) within 16 h of light at 25°C and 8 h of darkness at 21°C with constant 60% relative humidity. Once plants developed a second pair of true leaves (approximately 6–7 days), they were separated into four groups: (1) control, (2) chitosan treatment, (3) *A. rolfsii* inoculation, and (4) chitosan priming, followed by *A. rolfsii* inoculation (chitosan + *A. rolfsii*). Chitosan priming was carried out as described below (*Day 6*). Eight replicates were performed for control and chitosan treatment conditions, while 12 replicates were performed for *A. rolfsii* inoculation and chitosan + *A. rolfsii* conditions.

#### 2.5.2 Chitosan priming (Day 6)

In chitosan conditions, plain Hoagland solution was substituted with .2% w/v colloidal chitosan, dispersed in a Hoagland solution. In control and *A. rolfsii* inoculation conditions, Hoagland solution was replaced by the fresh solution. Plants were incubated under the same condition for another 2 days.

#### 2.5.3 *A. rolfsii* inoculation (Day 8) and plant sample collection (Day 13)

The *A. rolfsii* strain BRIP 39302a was cultured on PDA in a Petri dish. One week after, a plate full of mycelia was used for plant inoculation. The mycelia were incised into a square disc of 5 × 5 mm and placed inside the Root‐TRAPR system, adjoining the plant crown and floating on the surface of hydroponic solution as shown in Figure S1 (*Day 8*). Plants were infected for 5 days, and plant samples were collected for shoot, root tissues and exudate, and stored at −80°C until analysis (*Day 13*).

To confirm the organism caused disease, upon sample collection (*Day 13*), small parts of stem and root tissues were excised, surface sterilized in .04% sodium hypochlorite for 1 min, and then cultured on a PDA plate supplied with antibiotics, rifampicin (10 μg mL^−1^) and chloramphenicol (30 μg mL^−1^). The plate was incubated at room temperature for 7 days and fungal morphology was then observed.

### 2.6 *A. rolfsii* ITS DNA amplification and sequencing

Fungal DNA was extracted from mycelium using cetyltrimethylammonium bromide (CTAB) buffer extraction method as described previously (Pitkin et al., 1996). The internal transcribed spacer (ITS) regions were amplified with primers ITS1 (5′‐TCCGTAGGTGAACCTGCGG‐3′) and ITS4 (5′‐TCCTCCGCTTATTGATATGC‐3′). PCR was performed using *Ex Taq* DNA polymerase (TaKaRa Bio, Japan) according to the manufacturer's protocol using a T100 thermal cycler (Bio‐Rad, USA). A 20 μL aliquot of the amplification product was resolved in 1% agarose gel electrophoresis and stained with ethidium bromide. The gel band at approximately 700 bp was excised and extracted using the Qiaquick Gel Extraction kit (Qiagen, Germany) according to the manufacturer's protocol. The PCR product was directly subjected to the Sanger DNA sequencing, but the chromatogram data showed heterogeneities of nucleotides in several positions.

Therefore, PCR products were cloned into plasmids to achieve single copies of DNA. The plasmids were constructed using the TOPO TA Cloning kit (Invitrogen, USA) and transformed into NEB 5‐alpha competent *Escherichia coli* (New England BioLabs, USA). Transformed bacteria were recovered in Super Optimal broth with Catabolite repression (SOC), plated and grown at 37°C overnight on Luria–Bertani (LB) agar, containing X‐Gal and isopropyl β‐d‐1‐thiogalactopyranoside (IPTG). White colonies were picked, diluted in Terrific Broth (TB) containing 50 μg mL^−1^ kanamycin and incubated at 37°C overnight. Plasmid DNA was extracted using QIAprep Spin Miniprep kit (Qiagen, Germany) and digested with restriction enzymes, EcoRI and TaqαI (New England BioLabs, USA), using the manufacturers' protocols. The digested products were resolved in an agarose gel electrophoresis and stained with ethidium bromide. The products showing corresponding bands consistent with the ITS size (approximately 700 bp) were subjected to the Sanger DNA sequencing at the Australian Genome Research Facility (AGRF), Melbourne.

### 2.7 Sequence alignment and phylogenetic tree construction

Sequencing resolved two copies of the ITS sequences, deposited in the GenBank NCBI database (accessions OP369074 and OP369075). The records of other *A. rolfsii* ITS sequences were retrieved from the database. This included two strains isolated from *C. sativa* and 10 strains isolated from other plant hosts. All sequences were aligned using ClustalW tool within the MEGA11 software. Phylogenetic trees were built using Maximum‐likelihood and their robustness was accessed with 1000 bootstraps. ITS sequence of *Sclerotium hydrophilum* was used as an outgroup.

### 2.8 Shoot and root‐growth measurement

Plant height, leaf lengths and number of leaves were manually measured throughout the study. Root phenotype was scanned using an optical scanner (Epson Perfection V800, Japan) and analyzed using the WinRHIZO Arabidopsis 2019 program (Regent Instruments, Canada). Root length and surface area were calculated using the method previously described (Suwanchaikasem et al., 2022). Shoot and root fresh weights (FWs) were measured on the collection day using an analytical balance (Ohaus, USA).

### 2.9 Peroxidase and chitinase activity assays

Approximately 100 mg of shoot and root tissue samples were extracted with 400 μL of 100 mM phosphate buffer, pH 6.5. Exudate solution was concentrated with an Amicon Ultra‐15, 10 kDa molecular weight cutoff (MWCO) device (Merck Millipore, Germany) from approximately 14 mL starting volume to yield approximately 200 μL of protein concentrate according to the previous method (Suwanchaikasem et al., 2022). Total peroxidase and chitinase activities were measured using the methods described previously (Suwanchaikasem et al., 2022). For peroxidase activity, protein extracts were mixed with .025% H_2_O_2_ and 50 mM guaiacol and the rate of absorbance change within 3 min was detected at 470 nm. For chitinase activity, protein extracts were treated with 1% colloidal chitin for 2 h and pH adjusted using 1 M sodium borate buffer, pH 8.5. Acidic dimethylaminobenzaldehyde (DMAB) reagent was added to colorize the released *N*‐acetyl‐d‐glucosamine (*N*‐GlcNAc) product. Absorbance was measured at 585 nm and compared with a *N*‐GlcNAc standard curve (50–2000 nM). FW was used for normalizing the data measured from shoot and root tissues. Root surface area (RSA) was applied to the measurement from root exudate.

### 2.10 Phytohormone detection

Phytohormones were extracted from the tissue samples using 200 μL of 70% methanol supplied with 500 ng mL^−1^ of six internal standards ([^2^H]_5_‐zeatin, [^2^H]_2_‐indole‐3‐acetic acid [IAA], [^2^H]_7_‐cinnamic acid [CA], [^2^H]_4_‐SA, [^2^H]_6_‐abscisic acid [ABA], and dihydro‐JA). The analysis was performed using the Triple‐Quad 6410 LC–MS machine (Agilent Technologies, USA) equipped with Poroshell 120 EC‐C18 column (2.7 μm; 2.1 × 100 mm, Agilent Technologies, USA). The LC and MS parameters were set as described previously (Suwanchaikasem et al., 2023).

### 2.11 LC–MS/MS analysis of exudate proteome

After concentration using an Amicon Ultra‐15, 10 kDa MWCO centrifugal device (Merck Millipore, Germany), 100 μL of concentrated exudate proteins (approximately 5–11 μg protein) were aliquoted from each sample and processed with the S‐Trap micro spin column (Protifi, USA) using the protocol previously described (Suwanchaikasem et al., 2023). Final peptides were loaded into the nano‐LC–MS/MS system.

The nano‐LC system, Ultimate 3000 RSLC (Thermo Fisher Scientific, USA) was equipped with the Acclaim Pepmap RSLC analytical column (C18, 100 Å, 75 μm × 50 cm) and Acclaim Pepmap nano‐trap column (C18, 100 Å, 75 μm × 2 cm). The column temperature was 50°C. Mobile phases A and B were .1% formic acid (FA) + 5% dimethyl sulfoxide (DMSO) in water and ACN, respectively. Injection volume was 6 μL. The trap column was loaded with peptide sample at an isocratic flow of 2% ACN containing .05% trifluoroacetic acid (TFA) at 6 μL min^−1^ for 5 min, followed by the switch of the trap column as parallel to the analytical column. LC flow rate was 300 nL min^−1^, and gradient was set as follows: 2% to 5% B (0–5 min), 5% to 24% B (5–75 min), 24% to 35% B (75–83 min), 35% to 85% B (83–84 min), 85% B (84–86 min), 85% to 2% B (86–87 min), and 2% (87–95 min). The timsTOF Pro MS (Bruker, Germany) was operated in parallel accumulation serial fragmentation (PASEF) mode. Data‐dependent acquisition (DDA) was performed with 10 PASEF MS/MS scans per cycle with a cycle time of 1.17 s. The electrospray ionization voltage was 1.6 kV, and drying gas flow rate and temperature were 3 L min^−1^ and 180°C. Mass range for both MS and MS/MS scans was 100–1700 *m/z*. Ion mobility resolution was .6–1.6 V·s cm^−1^ over a ramp time of 100 ms. A polygon filter was applied in the *m/z* and ion mobility space to exclude low *m/z*, single charged ions from PASEF precursor selection. An active exclusion time of .4 min was applied to precursors that reach 20 k intensity units. Collision energy was ramped from 20 to 59 eV along with increased ion mobility from .6–1.6 V·s cm^−1^.

### 2.12 Data processing of exudate proteome

Raw LC–MS/MS data were loaded to MaxQuant version 2.0 software and searched against *C. sativa* plant and *A. rolfsii* fungal protein databases. The *C. sativa* database was created by retrieving all *C. sativa* proteins from the NCBI web service. The *A. rolfsii* database was created from the genome sequence of *A. rolfsii* isolate ZY, genome accession JABRWG01 (Yan et al., 2021). Protein prediction was performed using AUGUSTUS bioinformatics web server (https://bioinf.uni-greifswald.de/webaugustus/). The prediction parameters were set as default, and *Phanerochaete chrysosporium* was selected as a species identifier due to its closest relationship to *A. rolfsii*. MaxQuant protein search parameters and data filtering were set as described previously (Suwanchaikasem et al., 2023), with additional filtering criteria that the included proteins must be identified from at least half of the total number of replicates from at least one sample group. Data acquisition was applied without quantitative methods selected. The final proteomic results were normalized to the initial volume of exudate collected, approximately 14 mL each.

In total, 38 *C. sativa* and 48 *A. rolfsii* protein groups were identified from the dataset, and protein identification details are reported in Table S1. The protein number (CS_01–CS_38 and AR_01–AR_48) was ranked based on total signal intensity from the highest to the lowest value. The intensity data were square root (SQRT) transformed to reduce right skewness and generate a homoscedastic dataset. The SQRT‐transformed data were used for constructing principal component analysis (PCA), partial least squares discriminant analysis (PLSDA), and hierarchical clustering heatmap using online MetaboAnalyst version 5.0 software (https://www.metaboanalyst.ca/). The PCA and PLSDA plots were built from all 86 proteins identified. The heatmap was constructed based on 58 significant proteins.

Because all 48 *A. rolfsii* proteins were identified against an in‐house protein database, protein sequences were then aligned against the NCBI database using the BLASTp tool to annotate protein names. Non‐redundant standard database of fungal taxa (ID 4751) was selected for the alignment. The closest matched protein, showing the highest alignment score, expected value (*E* value), and percent identity, with characterized protein name was reported (Table S1).

### 2.13 Statistical analysis

One‐way ANOVA, followed by Tukey's post hoc analysis was applied to test null hypothesis (*p* < .05) for all analyses, except exudate proteome data, using Minitab Statistical software version 20.3 (Minitab, USA). For proteomics data, statistical analysis was performed using one‐way ANOVA with permutation‐based false discovery rate (FDR) of 250 randomizations followed by Tukey's post hoc test (*q* < .05) using Perseus version 2.0 software.

## 3 RESULTS

Four different fungi (*A. rolfsii*, *M. phaseolina*, *Fusarium* sp., and *Phomopsis* sp.) isolated from diseased *C. sativa* were sourced from the Biological Collections, Queensland, and subsequently tested for *C. sativa* infection in in vitro hydroponic conditions using the Root‐TRAPR plant growth device. All fungal strains were introduced to the plant in the same manner, adjacent to the plant crown and floating on the plant–solution interface (Figure S1). The *A. rolfsii* was the only strain causing disease in a robust manner within this experimental setup (Figure S2). Records also show that *A. rolfsii* is a prominent disease‐causing agent on *C. sativa* (Amaradasa et al., 2020; Chatzaki et al., 2022; Punja, 2021; Singh et al., 2022). Therefore, this strain was chosen to perform the plant‐pathogen experiments in this study to examine the pathological effect on *C. sativa* and the priming effect of chitosan to prevent the infection.

### 3.1 Confirmation of strain BRIP 39302a as *A. rolfsii* and a pathogen of *C. sativa*

The species designation of strain BRIP 39302a was confirmed by ITS amplification and sequencing. Two different copies (submitted to GenBank as accessions OP369074 and OP369075) were identified from the isolate. The sequences were aligned with other *A. rolfsii* sequences in the database, and a phylogenetic tree was built. The tree was separated into two main branches, where the two ITS copies of BRIP 39302a fell into both clusters (Figure S3), confirming the fungal species as *A. rolfsii* and revealing two main variations of the ITS gene within this species.

At the end of the plant inoculation experiments, small parts of stem and root tissues were excised, surface sterilized, and then cultured on PDA media. Within 7 days, the cultures from control and chitosan‐treated plants showed no sign of fungal growth (Figure S4), whereas the cultures from both *A. rolfsii* inoculation conditions (with and without chitosan priming) showed a white, fluffy, fan‐shaped fungal growth that later developed light brown sclerotia, which was identical to the culture before inoculation (Figure S4), confirming *A. rolfsii* was the cause of disease. As the fungus was introduced to the plant crown, a connection between stem and root, the pathogen was expected to penetrate both upward and downward to infect plant shoot and root systems. However, only stem tissues showed fungal growth from the tissue isolates. Root tissues were clean, implying that the pathogen might not progress into plant roots within this hydroponic setup.

### 3.2 .2% w/v chitosan neither inhibits nor promotes *A. rolfsii* growth

**FIGURE 1:**
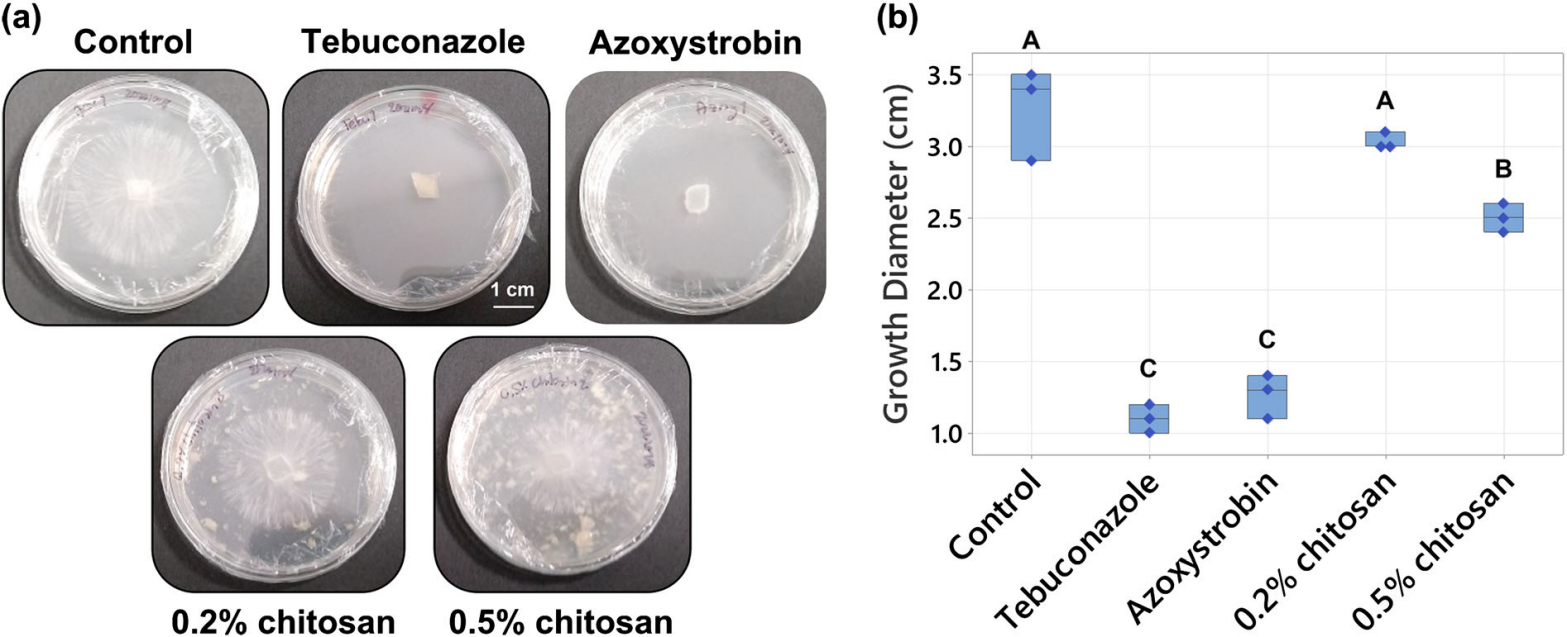
Testing the effects of chitosan and antifungal agents on *Athelia rolfsii* growth. (a) Representative images and (b) box plot of fungal growth on the PDA plates for control, .2% and .5% chitosan, compared with commercial fungicides, tebuconazole, and azoxystrobin (10 μg mL^−1^). White particles observed in chitosan conditions were insoluble parts of chitosan after dispersion in PDA media. Three biological replicates were performed per condition. Letters (A–C) refer to statistically significant difference (*p* < .05) using one‐way ANOVA, followed by Tukey's post hoc analysis.

Chitosan has been reported with fungicidal activity against several fungal pathogens including *A. rolfsii* (Eweis et al., 2006). Therefore, an in vitro antifungal assay was carried out to test the direct effect of chitosan against the pathogen at the effective concentrations to trigger plant defenses, .2% and .5% w/v (Suwanchaikasem et al., 2023). Within 3 days, *A. rolfsii* expanded to 3.27 ± .15 cm in diameter in control conditions (Figure 1a,b). Two commercial antifungal agents used as controls, tebuconazole that targets sterol synthesis and azoxystrobin that impairs mitochondrial function, showed a strong inhibitory effect. Fungal growth diameters in the media supplied with tebuconazole and azoxystrobin were only 1.10 ± .05 cm and 1.27 ± .07 cm, respectively (*p* < .001). The higher chitosan concentration (.5%) slightly inhibited *A. rolfsii* growth (2.50 ± .05 in diameter, *p* = .002), but the lower .2% chitosan had no significant impact on fungal development (3.03 ± .03 cm in diameter, *p* = .502).

Chitosan is a natural polysaccharide, made of glucosamine and *N*‐GlcNAc subunits. It is also a cell wall component of certain fungal species, especially members in Basidiomycota and Mucoromycotina divisions (Brown et al., 2020). Therefore, exogenous chitosan could be a resource for fungal growth. In this study, chitosan was examined alongside glucose using the YNB minimal nutrient media, containing nitrogen source and trace elements without any carbon source. As shown in Figure S5, growth of *A. rolfsii* in the YNB media, supplied with .2% and .5% glucose were denser and wider than the growth in YNB media without a carbon source. In contrast, growth of *A. rolfsii* in the YNB media, supplied with .2% and .5% chitosan were not different to the media without carbon. The mycelia were pale and expanded to a limited area, indicating that chitosan was not consumed, or only done so poorly, by the fungus (Figure S5).

Overall, the data indicates that .2% w/v colloidal chitosan had no direct effects, neither positive nor negative, toward *A. rolfsii* growth. Hence, .2% chitosan concentration was selected for the following plant‐pathogen experiments to trigger *C. sativa* defense responses.

### 3.3 *A. rolfsii* infects *C. sativa* shoot tissues and chitosan inhibits root development

**FIGURE 2:**
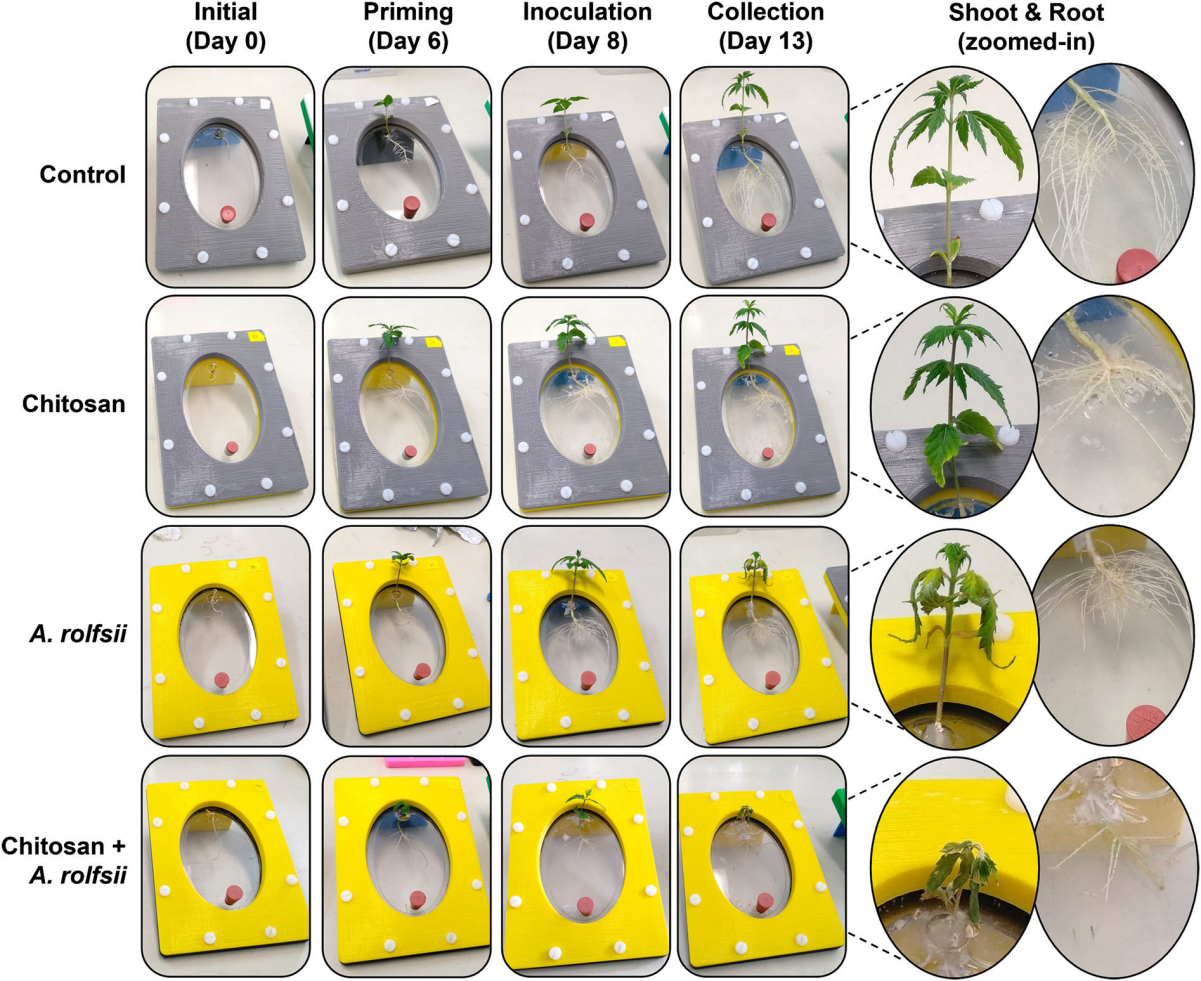
Morphology of *Cannabis sativa* plants in the Root‐TRAPR system. Four different groups consisting of (1) control, (2) chitosan treatment, (3) *Athelia rolfsii* inoculation, and (4) chitosan priming with *A. rolfsii* inoculation (chitosan + *A. rolfsii* ) conditions are shown in each row. In column, four timepoints are *initial (Day 0)*: the first day when plant seedings were transferred to the Root‐TRAPR system, *priming (Day 6)*: 6 days later when chitosan was introduced to plant roots, *inoculation (Day 8)*: 2 days after priming when *A. rolfsii* pathogen was introduced the system, and *collection (Day 13)*: 5 days after inoculation when plant samples were collected. The insets on the right show not‐to‐scale zoomed‐in images of shoot and root morphology among four conditions on the collection day (*Day 13*).

**FIGURE 3:**
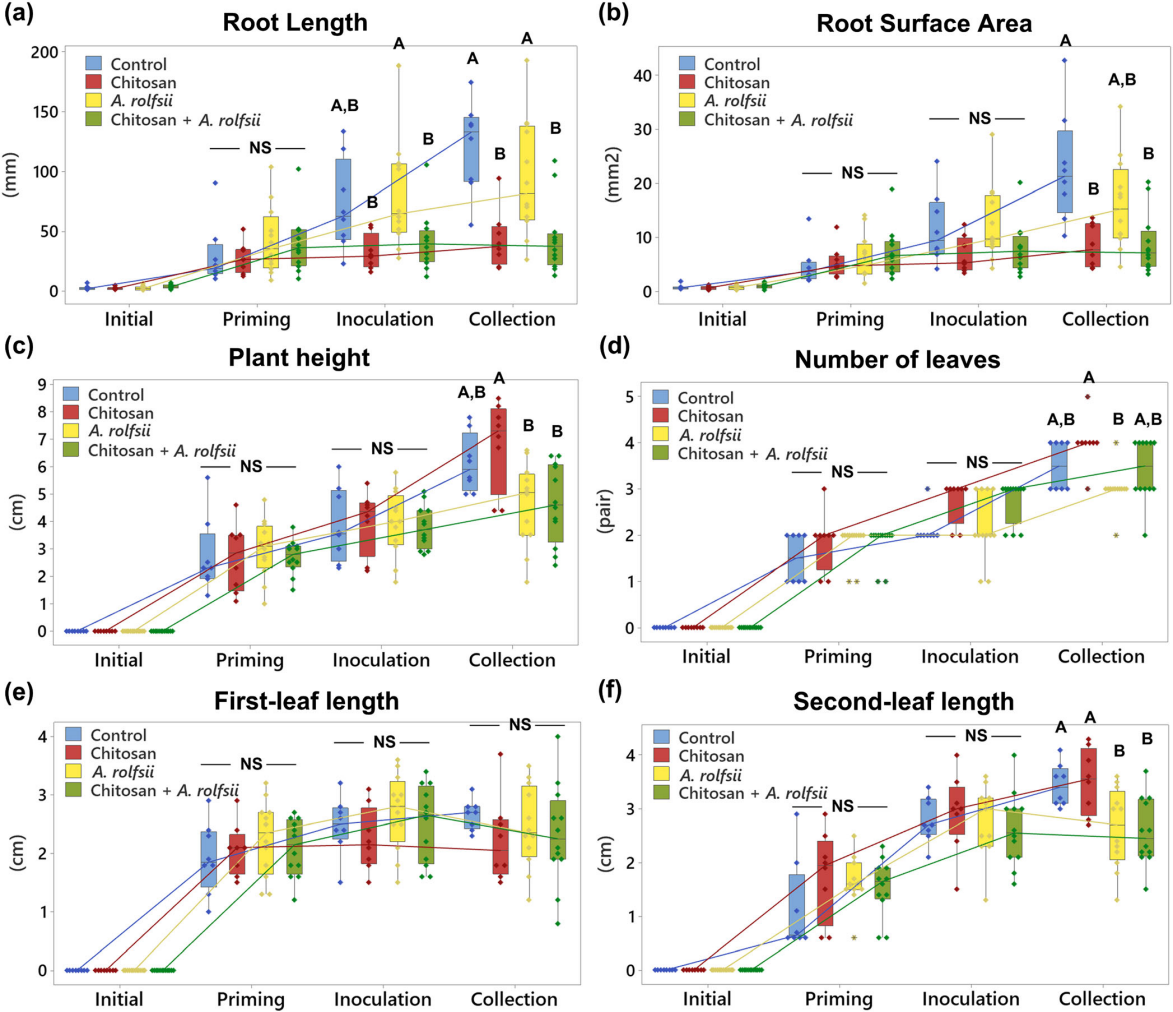
Measurement of *Cannabis sativa* root and shoot growth in response to chitosan and inoculation with *Athelia rolfsii* . Two root parameters are (a) root length and (b) root surface area. Four shoot parameters consist of (c) plant height, (d) number of leaves, (e) first‐leaf length, and (f) second‐leaf length. All parameters were measured from four conditions: (1) control, (2) chitosan treatment, (3) *A. rolfsii* inoculation, and (4) chitosan + *A. rolfsii* condition, at four timepoints: *initial (Day 0)*, *priming (Day 6)*, *inoculation (Day 8)*, and *collection (Day 13)*. Eight replicates were performed for control and chitosan conditions, and 12 replicates were performed for *A. rolfsii* inoculation and chitosan + *A. rolfsii* conditions. The data are depicted in boxplots, showing interquartile range box, whiskers, median, and outliers with a median connect line between each timepoint within the same conditions. Letters (A and B) refer to statistically significant difference (*p* < .05) across four conditions within the same day using one‐way ANOVA, followed by Tukey's post hoc analysis. NS refer to a non‐significant difference (*p* ≥ .05) using one‐way ANOVA across four sample groups.

After 6 days in the Root‐TRAPR system, one set of *C. sativa* roots was treated with .2% colloidal chitosan. Two days after the treatment (*Day 8*), interruption of root development upon chitosan treatment was observed (Figure 2), where root length and surface area of chitosan treatment group were 45.54 ± 6.73% (*p* = .109) and 57.29 ± 9.36% (*p* = .300) of the control, respectively (Figure 3a,b and Table S2). The effects were significantly different from control at the collection time (*Day 13*), when root length and surface area were 35.65 ± 6.62% (*p* = .001) and 37.25 ± 4.60% (*p* = .003) of the control, respectively. However, shoot growth was unaffected by root chitosan treatment, plant height (*p* = .765), number of leaves (*p* = .283), first‐leaf length (*p* = .605), and second‐leaf length (*p* = .995) of chitosan‐treated plants were comparable with the control at the collection point (Figure 3c–f and Table S2).

Two days after chitosan treatment (*Day 8*), *A. rolfsii* was introduced to the plant crown. The inoculation was applied to two groups including *A. rolfsii* inoculation and chitosan + *A. rolfsii* conditions. Five days after inoculation (*Day 13*), *A. rolfsii* highly affected *C. sativa* shoot growth but less so for root development. Shoots of the inoculated plants showed wilt symptoms, that is, drooping leaves and branches and yellowing leaves with blackening spots (Figure 2). The second‐leaf length of *A. rolfsii*‐inoculated plants was significantly shorter than control by 23.39 ± 5.93% (*p* = .036) (Figure 3f). However, root symptoms, such as root rot or root browning, occasionally reported for southern blight disease in other crops (Mullen, 2001), were not observed on the inoculated plants in this study. Root length and surface area of the inoculated plants were slightly smaller than control by 21.55 ± 11.17% (*p* = .433) and 29.10 ± 10.29% (*p* = .223), respectively (Figure 3a,b and Table S2).

Based on morphological observation, chitosan‐primed infected plants (chitosan + *A. rolfsii*) showed no indication of disease resistance. The plants still suffered from the disease and showed visible disease symptoms of yellowish and wilted leaves (Figure 2). On the collection day (*Day 13*), plant height (*p* = 1.000), number of leaves (*p* = .266), first‐leaf length (*p* = .960), and second‐leaf length (*p* = .994) were not different from the unprimed infected plants (Figure 3c–f and Table S2). In turn, chitosan‐primed infected plants had significantly shorter root length and smaller root surface area as compared with the unprimed infected plants. The root length and surface area were only 46.54 ± 8.68% (*p* = .011) and 54.45 ± 9.55% (*p* = .090) of the infected plants, respectively (Figure 3a,b and Table S2), which was the result of root exposure to chitosan.

### 3.4 Shoot defense enzyme activities are activated upon *A. rolfsii* infection and root chitosan treatment reduces shoot peroxidase activity

**FIGURE 4:**
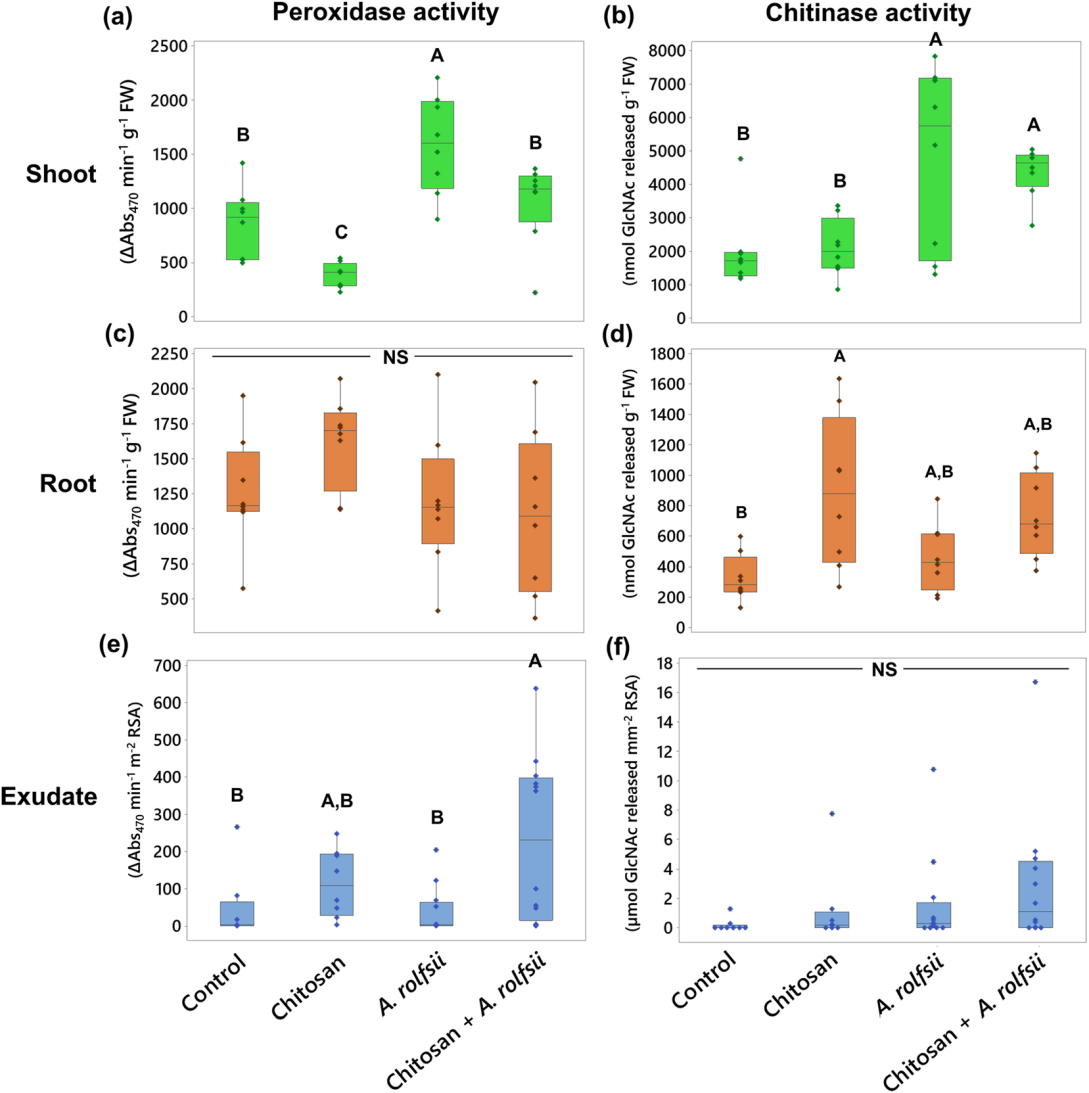
Total peroxidase and chitinase activities measured from (a,b) shoot, (c,d) root tissues, and (e,f) exudates across four groups: (1) control, (2) chitosan treatment, (3) *Athelia rolfsii* inoculation, and (4) chitosan + *A. rolfsii* conditions. Eight replicates were performed per condition. The data are depicted in boxplots, showing interquartile range box, whiskers, median, and outliers. Letters (A and B) refer to statistically significant difference (*p* < .05) using one‐way ANOVA, followed by Tukey's post hoc analysis. NS refers to a non‐significant difference (*p* ≥ .05) across four sample groups.

In Figure 4a,b and Table S2, total peroxidase and chitinase activities measured from the shoot tissues of *A. rolfsii*‐infected plants were both significantly higher than those of control by 1.85 (*p* = .001) and 2.43 times (*p* = .006), respectively, meaning that defense enzymes were activated in shoot tissues of the infected plants to counteract the pathogen infection. In chitosan treatment alone, total peroxidase activity was lower than control by 2.22 times (*p* = .047), but total chitinase activity was unaffected (*p* = .999), suggesting root chitosan treatment might suppress shoot peroxidase activity. In chitosan priming condition (chitosan + *A. rolfsii*), total peroxidase activity was significantly lower than that of the infected plants by approximately 1.5 times (*p* = .021) and relatively comparable with control (*p* = .665), implying shoot peroxidase activity of the chitosan‐primed plants was not activated to the level observed in the unprimed infected plants (Figure 4a). In contrast, total chitinase activity of the chitosan‐primed infected plants was nearly comparable with the unprimed infected plants (*p* = .934) but significantly higher than that of control (*p* = .027) and chitosan treatment alone (*p* = .037), suggesting that *A. rolfsii* infection was the main factor inducing total chitinase activity in *C. sativa* shoot tissues, but root chitosan treatment had no effect in this regard (Figure 4b).

### 3.5 Root defense enzyme activities are not affected by *A. rolfsii* infection but promoted by chitosan treatment

Total peroxidase activity measured from the root tissues was comparable across all four conditions, suggesting the pathogen and chitosan treatment had no impact on root peroxidase activity (Figure 4c and Table S2). In contrast, total chitinase activity was promoted by chitosan treatment. Total chitinase activities of chitosan treatment and chitosan + *A. rolfsii* conditions were 2.72 (*p* = .007) and 2.27 times (*p* = .066) higher than control, respectively. However, the activity measured from *A. rolfsii*‐infected plants without chitosan priming was comparable with control (*p* = .827) (Figure 4d and Table S2).

In exudate, total peroxidase activity was likely enhanced by chitosan treatment (Figure 4e and Table S2). The highest activity was observed from chitosan + *A. rolfsii* condition with 5.03 times higher than that of control (*p* = .027). The activity was 2.47 times higher than the control in the chitosan treatment condition (*p* = .763) but 1.22 times lower than the control in the *A. rolfsii* infection condition (*p* = .999). Total chitinase activity was comparable across all four conditions (ANOVA *p* = .324). Nonetheless, peroxidase activity was detected with high variation in all sample groups, and chitinase activity was very low or undetectable in several replicates (Figure 4f and Table S2). For example, five exudate samples of chitosan + *A. rolfsii* condition had peroxidase activity below 100 ΔAbs_470_ min^−1^ m^−2^ RSA, but six samples had the activity above 300 ΔAbs_470_ min^−1^ m^−2^ RSA. Six out of eight control samples had undetectable chitinase activity. Nine out of 12 *A. rolfsii*‐infected samples had chitinase activity below 1 μmol *N*‐GlcNAc released mm^−2^ RSA. Hence, further investigation is required to closely examine the peroxidase and chitinase activities in the exudates upon chitosan priming and *A. rolfsii* infection.

### 3.6 Shoot phytohormones and metabolites are induced upon *A. rolfsii* infection

**FIGURE 5:**
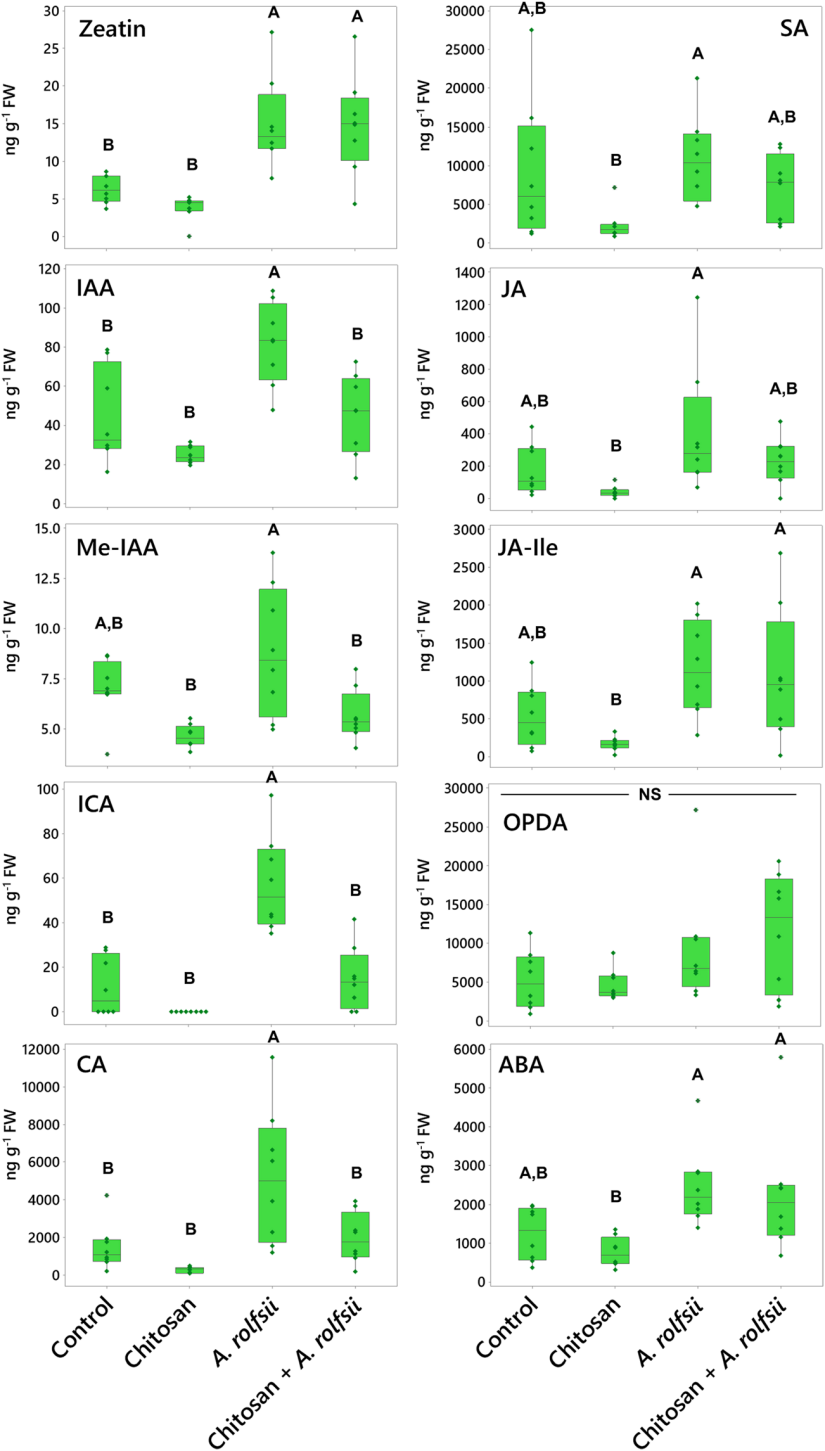
Phytohormone and metabolite levels measure from shoot tissues across four groups: (1) control, (2) chitosan treatment, (3) *Athelia rolfsii* inoculation, and (4) chitosan + *A. rolfsii* conditions. Eight replicates were performed per condition. The data are depicted in boxplots, showing interquartile range box, whiskers, median, and outliers. Letters (A and B) refer to statistically significant difference (*p* < .05) using one‐way ANOVA, followed by Tukey's post hoc analysis. NS refers to a non‐significant difference (*p* ≥ .05) across four sample groups. IAA, indole‐3‐acetic acid; Me‐IAA, methyl‐indole‐3‐acetic acid; ICA, indole‐3‐carboxylic acid; CA, cinnamic acid; SA, salicylic acid; JA, jasmonic acid; JA‐Ile, jasmonic acid‐isoleucine; OPDA, 12‐oxo‐phytodienoic acid; ABA, abscisic acid.

Some shoot phytohormone and metabolite levels were increased upon *A. rolfsii* infection (Figure 5 and Table S2). The levels of two metabolites detected, indole‐3‐carboxylic acid (ICA) and CA, were significantly increased by 5.24 (*p* < .001) and 3.51 times (*p* = .006), respectively. The levels of growth hormones, zeatin and IAA, were also significantly increased by 2.38 (*p* = .004) and 1.85 (*p* = .003) times, respectively. The levels of defense‐related hormones, JA, JA‐isoleucine (JA‐Ile), and ABA, showed increasing tendency, with 2.31 (*p* = .188), 2.16 (*p* = .164), and 1.98 (*p* = .103) times higher than control, respectively. In chitosan treatment without pathogen inoculation, shoot hormones and metabolites were slightly decreased. The maximum decreases were observed in CA, JA, and SA levels, with 5.12 (*p* = .651), 4.35 (*p* = .411), and 3.96 times (*p* = .107) lower than control, respectively. When comparing against the infected plants, CA, JA, and SA levels of chitosan‐treated plants were significantly lower by 17.98 (*p* < .001), 10.07 (*p* = .014), and 4.65 times (*p* = .033), respectively.

Interestingly, hormone and metabolite levels in chitosan priming condition (chitosan + *A. rolfsii*) were nearly comparable with control and not as high as those detected from the *A. rolfsii* infection condition (Figure 5 and Table S2). The levels of ICA and CA metabolites were significantly lower than those of the *A. rolfsii*‐infected plants by 3.87 (*p* < .001) and 2.63 times (*p* = .019), respectively, and not significantly different from control, with *p* = .949 and .962, respectively. The levels of auxins, IAA, and methyl‐IAA (Me‐IAA) were also significantly lower than those of the *A. rolfsii*‐infected plants by 1.81 (*p* = .004) and 1.56 times (*p* = .013), respectively, and not significantly different from control, with *p* = .999 and .526, respectively. Similar decreasing tendencies were also observed from the defense‐related hormones, SA and JA, even though statistically significant difference was not observed. The SA and JA levels of chitosan + *A. rolfsii* condition were 1.50 (*p* = .603) and 1.76 (*p* = .411) times lower than those of the unprimed infected plants, respectively, and nearly matched to the control (*p* = .899 and .958 for SA and JA, respectively). These results suggest that root chitosan treatment might mitigate the activation of hormones and metabolites in *C. sativa* shoots upon *A. rolfsii* infection.

### 3.7 Chitosan promotes accumulations of JA hormones and CA metabolite in root tissues but *A. rolfsii* infection affects root ABA level

**FIGURE 6:**
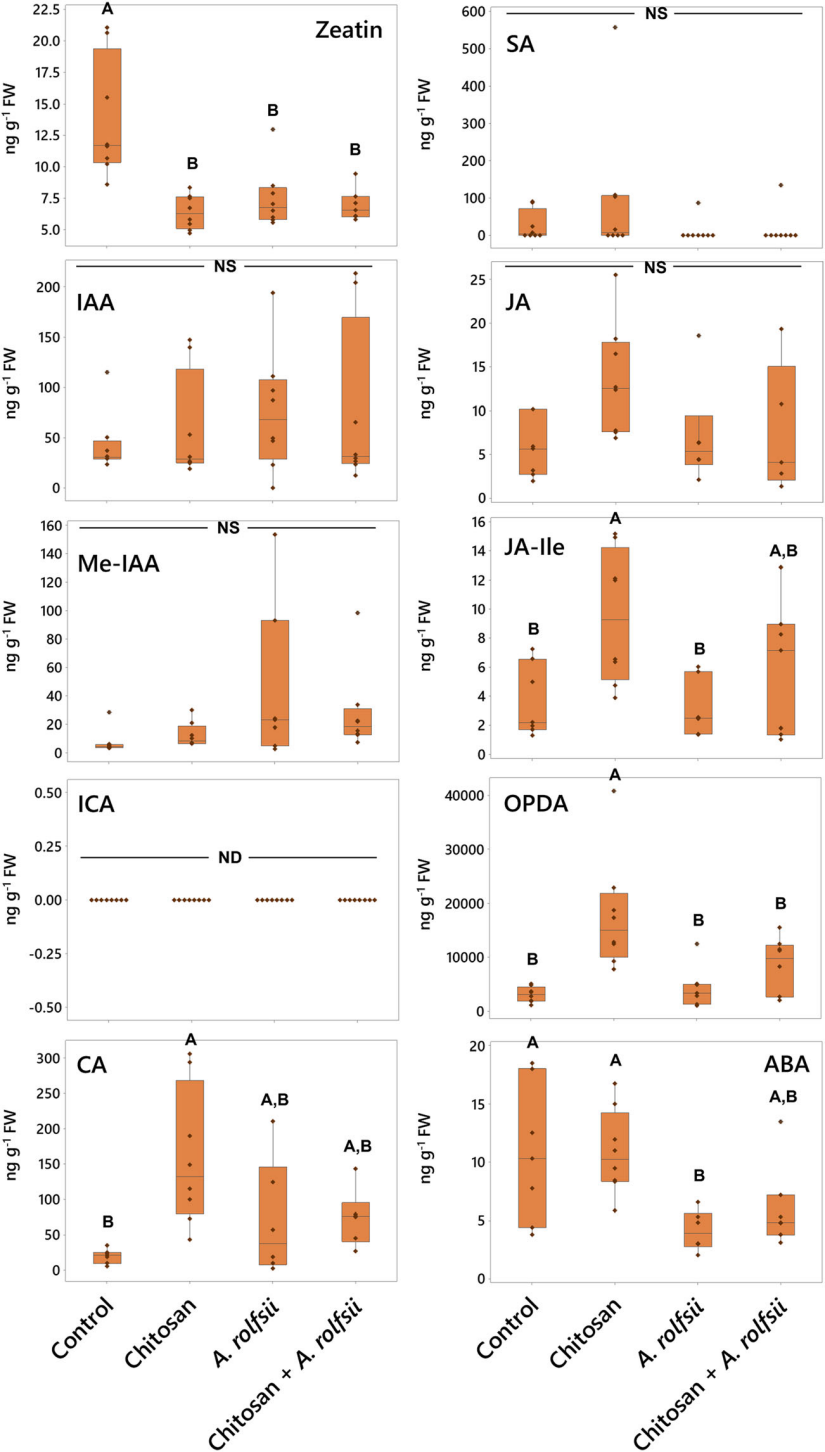
Phytohormone and metabolite levels in root tissues across four groups: (1) control, (2) chitosan treatment, (3) *Athelia rolfsii* inoculation, and (4) chitosan + *A. rolfsii* conditions. Eight replicates were performed per condition. The data are depicted in boxplots, showing interquartile range box, whiskers, median, and outliers. Letters (A and B) refer to statistically significant difference (*p* < .05) using one‐way ANOVA, followed by Tukey's post hoc analysis. NS refers to a non‐significant difference (*p* ≥ .05) across four sample groups. ND refers to a non‐detected data. IAA, indole‐3‐acetic acid; Me‐IAA, methyl‐indole‐3‐acetic acid; ICA, indole‐3‐carboxylic acid; CA, cinnamic acid; SA, salicylic acid; JA, jasmonic acid; JA‐Ile, jasmonic acid‐isoleucine; OPDA, 12‐oxo‐phytodienoic acid; ABA, abscisic acid.

In root tissues, phytohormone and metabolite levels were largely impacted by chitosan treatment (Figure 6 and Table S2). In chitosan‐treated plants, the level of CA metabolite was 7.94 times increased (*p* = .004). The levels of JA hormones and its derivatives, JA‐Ile and 12‐oxo‐phytodienoic acid (OPDA), were also increased, with 2.37 (*p* = .078), 2.55 (*p* = .026), and 5.76 times (*p* < .001) higher than control, respectively. These results demonstrate that chitosan treatment possibly promotes production and accumulation of JA hormones and CA metabolite in root tissues.

In *A. rolfsii* inoculation and chitosan priming (chitosan + *A. rolfsii*) conditions, two phytohormones, zeatin and ABA, were detected with significant decrease as compared with control (Figure 6 and Table S2). Zeatin levels were significantly decreased by 1.83 (*p* = .001) and 1.98 (*p* = .001) in *A. rolfsii* inoculation and chitosan priming conditions, respectively. ABA levels of both conditions were 2.61 (*p* = .034) and 1.78 times (*p* = .161) lower than that of control, respectively. Zeatin level in chitosan treatment was also significantly lower than that of control by 2.15 times (*p* < .001), but the ABA level was not changed (*p* = 1.000). This implies that root ABA levels might be affected by *A. rolfsii* infection but zeatin, one of the members in the cytokinin family, might be impacted by both chitosan treatment and *A. rolfsii* infection. The lower level of zeatin in root tissues of chitosan‐treated plants could also be related to the root‐growth interruption as observed from chitosan treatment (Figures 2 and 3).

### 3.8 Chitosan induces *C. sativa* to secrete defense proteins into exudate and *A. rolfsii* secretes cell wall‐degrading enzymes upon infection

**FIGURE 7:**
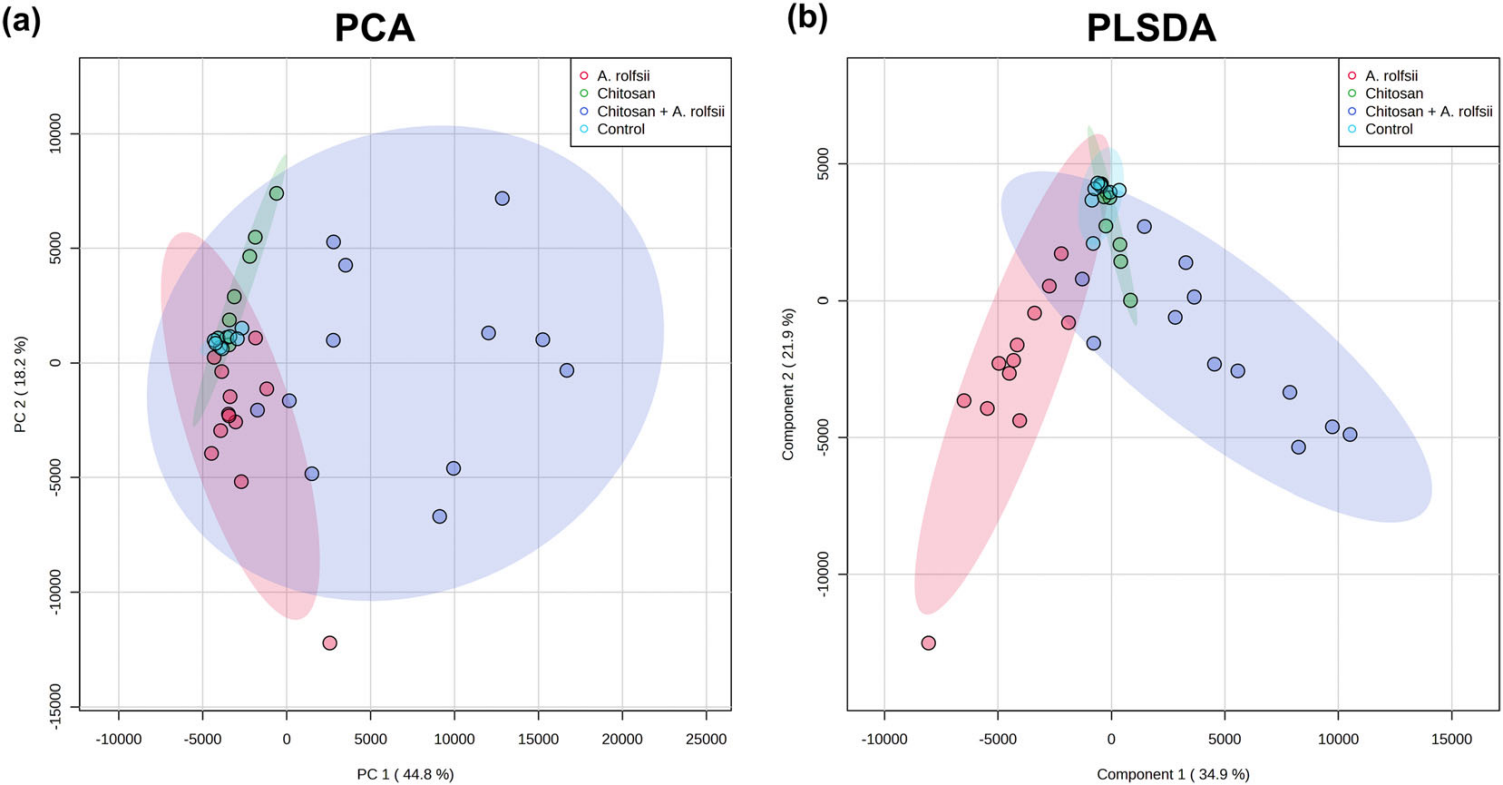
PCA (a) and PLSDA (b) plots of exudate proteomes across four conditions: (1) control, (2) chitosan treatment, (3) *Athelia rolfsii* inoculation, and (4) chitosan + *A. rolfsii* conditions. PC1 and PC2 are shown in *x* axis and *y* axis, respectively, and the colored 95% confidence ellipses were drawn around each sample group. Eight replicates of control and chitosan conditions and 12 replicates of *A. rolfsii* inoculation and chitosan + *A. rolfsii* conditions were analyzed.

One rationale for using the in vitro Root‐TRAPR hydroponic system in these experiments is that the collection of root exudates is achievable, and hence, the samples were easily managed and processed for proteomics analysis. Exudate proteomes of four experimental groups were characterized against *C. sativa* plant and *A. rolfsii* fungal databases. In total, 86 high‐confidence proteins, including 38 *C. sativa* and 48 *A. rolfsii* putative proteins, were identified from the entire dataset (Table S1). Protein profiles were largely different across four groups, influenced by both chitosan treatment and *A. rolfsii* infection. In PCA and PLSDA plots (Figure 7a,b), control samples (in cyan) were clustered closely together near zero origin. Chitosan‐treated samples (in green) were slightly separated from the control group, mainly in PC2 direction. *A. rolfsii*‐infected samples (in red) were shifted from the control in both PC1 and PC2 direction and away from the chitosan group. The samples of chitosan + *A. rolfsii* condition (in blue) were further separated from the control and stayed between the chitosan treatment and *A. rolfsii* infection groups, exhibiting a combining effect from both conditions.

**FIGURE 8:**
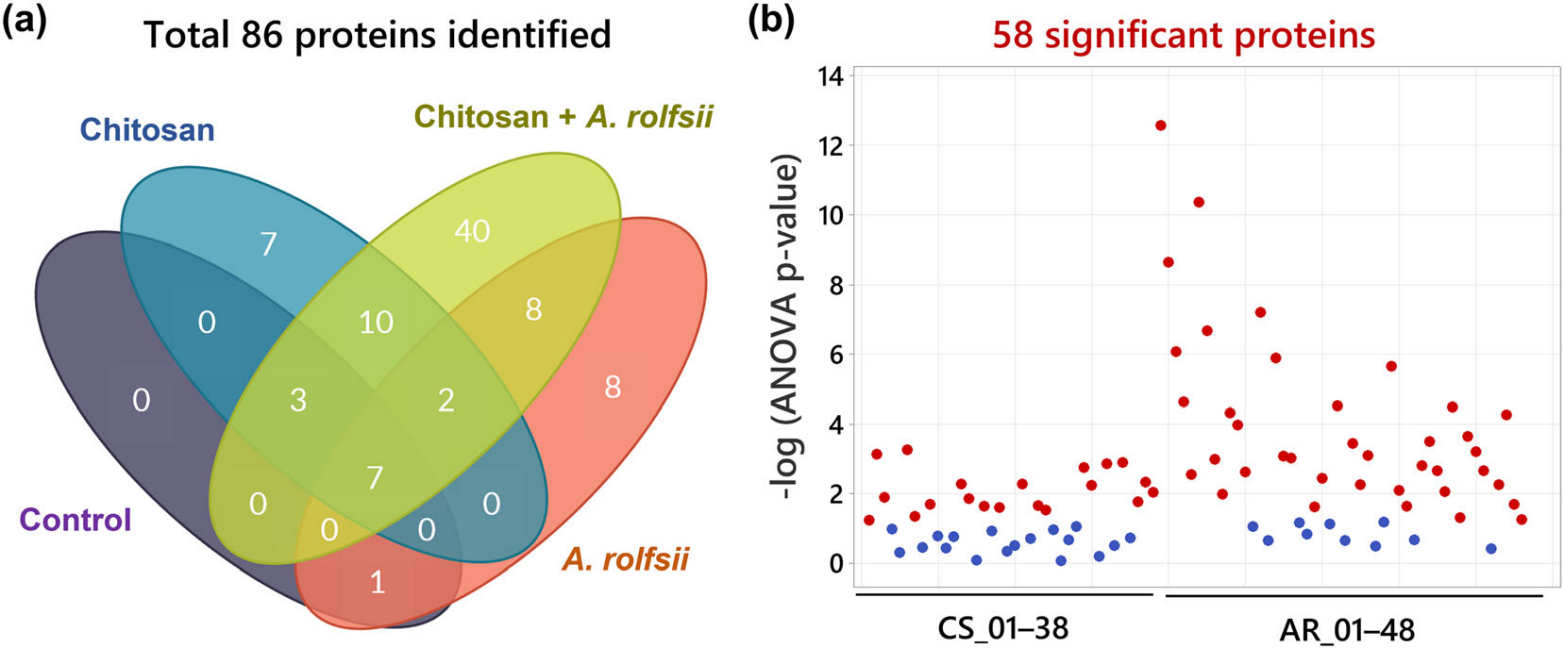
Qualitative data of all 86 proteins identified from the exudates. (a) Venn diagram showing number of proteins detected across four sample groups: control, chitosan treatment, *Athelia rolfsii* inoculation and chitosan + *A. rolfsii* conditions. The diagram was created using creately.com. (b) *p*‐value plot showing significant proteins (*q* < .05) as labeled in red of 20 *Cannabis sativa* (CS) and 38 *A. rolfsii* (AR) proteins. The protein number of each organism (CS_01–CS_38 and AR_01–AR_48) was ranked from the highest to the lowest total signal intensity. Protein identification details and statistical data are presented in Table S1.

Among 86 exudate proteins, only 11 proteins were identified from the control group. Most of them were plant cell membrane proteins, such as uclacynanin‐3, kiwellin, and cucumber peeling cupredoxin and ubiquitous intracellular proteins, such as actin, histone, and ubiquitin, which could be derived from sloughed dead cells (Table S1). These proteins were also detected in the other sample groups (Figure 8a), implying that they could be common proteins in *C. sativa* root exudate. Although there were 29 and 26 proteins detected from the chitosan treatment and *A. rolfsii* infection conditions, respectively, a large number of proteins, that is, 70 out of 86 proteins, were identified from chitosan + *A. rolfsii* condition, where 40 of them were specifically detected from this group (Figure 8a), indicating that the combination of chitosan elicitor and *A. rolfsii* pathogen enhanced the number of proteins secreted into the exudate.

**FIGURE 9:**
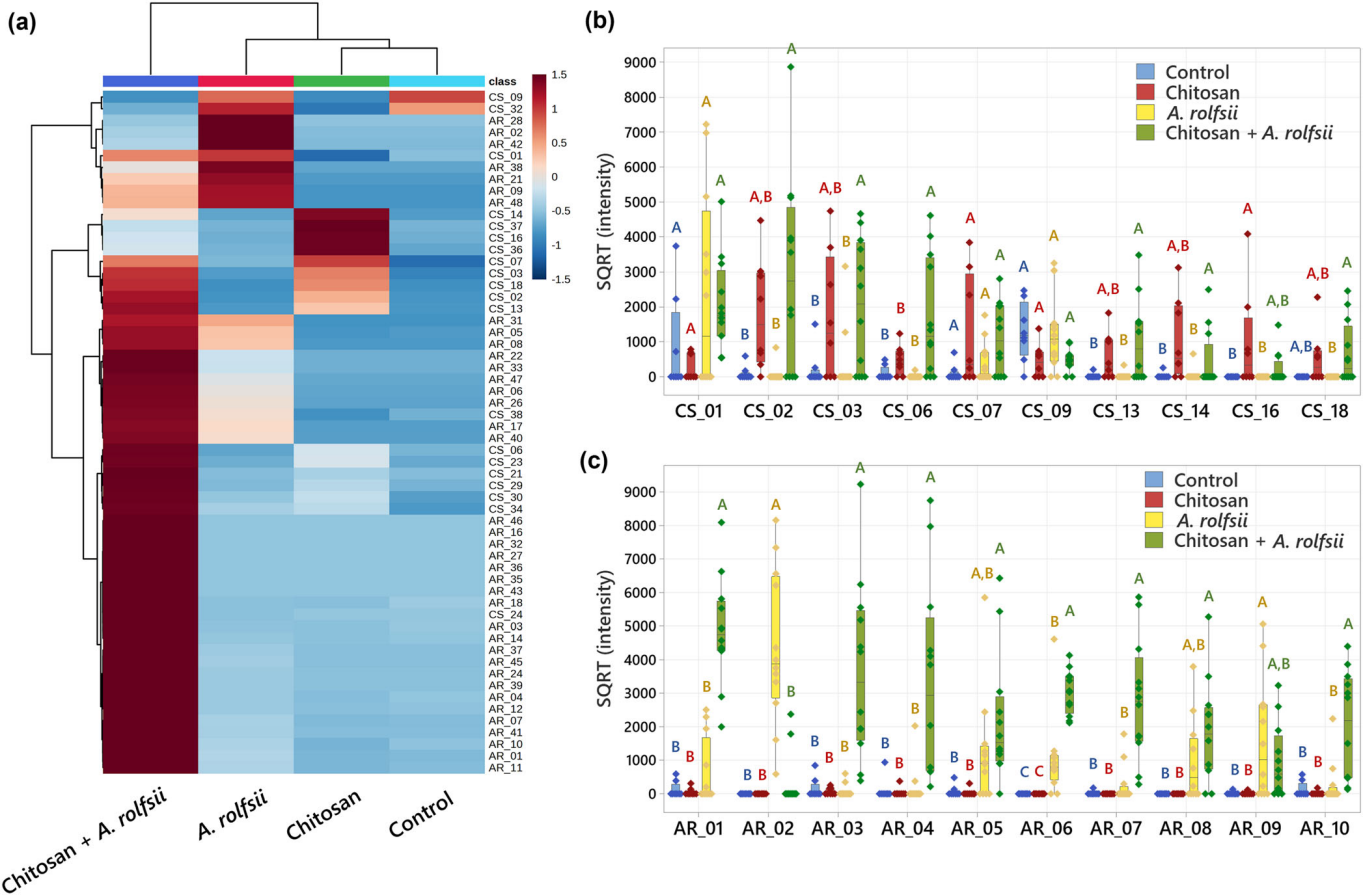
The interactions of *Cannabis sativa* , *Athelia rolfsii* , and chitosan alter root exudate protein secretion. (a) Heatmap analysis of all 58 significant proteins, showing sample clustering on horizontal axis and protein feature clustering on vertical axis. The heatmap was plotted from group averages of eight replicates of control and chitosan conditions and 12 replicates of *A. rolfsii* inoculation and chitosan + *A. rolfsii* conditions. The heatmap color was calculated using Euclidean distance method. (b) Intensity plots of top 10 highest abundant significant *C. sativa* (CS) proteins across four sample groups. (c) Intensity plots of top 10 highest abundant significant *A. rolfsii* (AR) proteins across four sample groups. (b,c) The data are depicted in boxplots, showing interquartile range box, whiskers, median, and outliers. Letters (A–C) refer to statistically significant difference (*q* < .05) using one‐way ANOVA with permutation‐based FDR, followed by Tukey's post hoc analysis. Full protein identification and statistical data are supplied in Table S1.

Statistical analysis (Figure 8b) highlighted that 20 out of 38 *C. sativa* (CS) and 38 out of 48 *A. rolfsii* (AR) proteins were significantly different across four experimental groups (*q* < .05). These 58 significant proteins were further analyzed to create a heatmap to provide an overview of changes (Figure 9a). There was no protein with significantly increased intensity in the control group. Proteins detected with significantly increased intensity in the chitosan treatment were all *C. sativa* proteins (Figure 9b) and likewise those with significantly increased level in the *A. rolfsii* infection were mostly *A. rolfsii* proteins (Figure 9c). In chitosan + *A. rolfsii* conditions, significant proteins were both *C. sativa* plant and *A. rolfsii* fungal proteins (Figure 9b,c). On the horizontal axis of the heatmap (Figure 9a), control and chitosan treatment groups were clustered together and *A. rolfsii* infection and chitosan + *A. rolfsii* condition were grouped on a separate branch, demonstrating that the overall exudate proteome profile of chitosan treatment was relatively closer to the control and the *A. rolfsii* pathogen caused larger change in the proteome profile than chitosan treatment. This heatmap result was well correlated with PCA and PLSDA results, displaying close relationship between exudate proteomes of control and chitosan sample groups and further separation of *A. rolfsii* infection and chitosan + *A. rolfsii* conditions (Figure 7a,b).

In the list of *C. sativa* significant proteins, many are plant defense proteins, for example, pathogenesis‐related (PR) protein R major‐form like (CS_02), thaumatin‐like protein 1 (CS_06), peroxidase 57‐like (CS_13), and peroxidase 24 (CS_16). Their intensities were significantly increased in chitosan treatment or chitosan + *A. rolfsii* condition (Figure 9b and Table 1), confirming the eliciting effect of chitosan to induce *C. sativa* roots to secrete defense proteins into exudate (Suwanchaikasem et al., 2023). For *A. rolfsii* proteins, their functions were tentatively assigned based on BLAST results, where the full protein sequence was aligned against the NCBI fungal protein repository and the first‐hit protein with the highest alignment score and annotated protein name was selected (Tables 1 and S1). In the list of *A. rolfsii* significant proteins, many are cell wall‐degrading enzymes, for example, exo‐beta‐1,3‐glucanase (AR_01), cellobiohydrolase (AR_03), endo‐1,4‐beta‐xylanase C precursor (AR_04), glucoamylase G2 (AR_05), and alpha‐amylase (AR_09). These glycoside hydrolase enzymes primarily function to degrade plant cell walls to allow pathogens to invade plant cells (Rafiei et al., 2021). Their intensities were increased in *A. rolfsii* infection or chitosan + *A. rolfsii* condition (Figure 9c and Table 1), suggesting that the pathogen secreted these digestive enzymes into exudate solution to attack *C. sativa* root tissues.

**TABLE 1:** Identification and statistical analysis of top 10 most abundant significant *Cannabis sativa* and *Athelia rolfsii* proteins.

Protein no.	Protein name	Protein ID	Sequence BLAST results				ANOVA *q* value	SQRT (intensity) (AU)^a^			
			Closely matched protein	Protein ID	Species	Identity		Control	Chitosan	*A. rolfsii*	Chitosan + *A. rolfsii*
*C. sativa* proteins											
CS_01	Mulatexin‐like	XP_030498669.1	‐	‐	‐	‐	.0459	834.2 ± 465.2	266.44 ± 122.16	2348.6 ± 782.0	2049.2 ± 360.6
CS_02	PR protein R major‐form like	XP_030501451.1	‐	‐	‐	‐	.0014	94.00 ± 68.8^B^	1796.4 ± 525.2^A,B^	69.1 ± 66.2^B^	2858.6 ± 764.7^A^
CS_03	HP G4B88_006669	KAF4382037.1	PR protein 1	XP_030487534.1	*C. sativa*	99.4%	.0113	219.9 ± 173.9^B^	1698.4 ± 604.1^A,B^	369.9 ± 263.3^B^	2031.5 ± 522.7^A^
CS_06	Thaumatin‐like protein 1	XP_030502231.1	‐	‐	‐	‐	.0016	104.9 ± 65.2^B^	471.4 ± 136.5^B^	15.5 ± 14.8^B^	1684.3 ± 466.5^A^
CS_07	UP LOC115709853	XP_030493949.1	Bowman–Birk type proteinase inhibitor 2 isoform X3	XP_030492663.1	*C. sativa*	48.0%	.0370	111.5 ± 81.7	1255.5 ± 528.3	400.2 ± 160.4	1103.1 ± 281.1
CS_09	Kiwellin	XP_030508145.1	‐	‐	‐	‐	.0171	1288.1 ± 279.1	446.4 ± 160.2	1209.7 ± 288.3	474.8 ± 84.8
CS_13	Peroxidase 57‐like	XP_030509376.1	‐	‐	‐	‐	.0055	24.8 ± 23.2^B^	559.5 ± 222.8^A,B^	27.7 ± 26.6^B^	1023.9 ± 322.2^A^
CS_14	Agglutinin‐1‐like	XP_030478853.1	‐	‐	‐	‐	.0120	31.6 ± 29.6^B^	1059.2 ± 380.5^A,B^	55.0 ± 52.7^B^	441.7 ± 234.0^A^
CS_16	Peroxidase 24	XP_030506673.1	‐	‐	‐	‐	.0180	.0 ± .0^B^	941.7 ± 477.9^A^	.0 ± .0^B^	223.8 ± 127.0^A,B^
CS_18	Ribosome‐inactivating protein cucurmosin	XP_030478848.1	‐	‐	‐	‐	.0182	.0 ± .0^A,B^	527.4 ± 257.5^A,B^	.0 ± .0^B^	670.2 ± 248.9^A^
*A. rolfsii* putative proteins											
AR_01	‐	g139.t1	Exo‐beta‐1,3‐glucanase	OCH86564.1	*Obba rivulosa*	67.1%	<.0001	121.0 ± 76.3^B^	55.3 ± 37.2^B^	650.1 ± 277.0^B^	4863.6 ± 440.7^A^
AR_02	‐	g7261.t1	Pectinesterase	XP_037226068.1	*Mycena indigotica*	57.0%	<.0001	.0 ± .0^B^	.0 ± .0^B^	4353.6 ± 636.3^A^	346.2 ± 226.2^B^
AR_03	‐	g4472.t1	Cellobiohydrolase	BAC81967.1	*A. rolfsii*	82.3%	<.0001	153.7 ± 102.5^B^	72.0 ± 34.2^B^	78.5 ± 52.8^B^	3632.0 ± 731.3^A^
AR_04	‐	g4320.t1	Endo‐1,4‐beta‐xylanase C precursor	XP_046084670.1	*Lentinula edodes*	60.4%	<.0001	116.9 ± 109.4^B^	47.0 ± 44.0^B^	200.1 ± 161.4^B^	3410.5 ± 797.9^A^
AR_05	‐	g4198.t1	Glucoamylase G2	BAA08436.1	*A. rolfsii*	96.1%	.0030	75.8 ± 56.2^B^	37.9 ± 35.4^B^	1222.0 ± 452.7^A,B^	2234.9 ± 528.3^A^
AR_06	‐	g6993.t1	HP K438DRAFT_1465487, partial	KAF8142613.1	*Mycena galopus*	73.6%	<.0001	.0 ± .0^C^	.0 ± .0^C^	1034.6 ± 332.3^B^	3032.6 ± 180.9^A^
AR_07	‐	g8683.t1	Exo‐glucanase 1	XP_047747642.1	*Psilocybe cubensis*	70.6%	<.0001	21.6 ± 20.2^B^	.0 ± .0^B^	264.5 ± 158.9^B^	2796.0 ± 499.2^A^
AR_08	‐	g10175.t1	HP PLICRDRAFT_57673	KII84270.1	*Plicaturopsis crispa*	55.7%	.0017	.0 ± .0^B^	.0 ± .0^B^	974.5 ± 338.4^A,B^	1847.7 ± 423.7^A^
AR_09	‐	g11083.t1	Alpha‐amylase	XP_007867350.1	*Gloeophyllum trabeum*	63.3%	.0087	17.0 ± 15.9^B^	14.2 ± 13.3^B^	1598.9 ± 496.8^A^	951.7 ± 303.5^A,B^
AR_10	‐	g1860.t1	Exo‐beta‐1,3‐glucanase	OCH86564.1	*O. rivulosa*	67.4%	<.0001	123.1 ± 76.6^B^	21.0 ± 19.6^B^	270.6 ± 182.4^B^	2014.4 ± 447.2^A^

Abbreviations: AU, arbitrary unit; HP, hypothetical protein; PR, pathogenesis related; SQRT, square root; UP, uncharacterized protein.

^a^ Letters (A and B) refer to statistically significant difference (*q* < .05) using one‐way ANOVA with permutation‐based FDR, followed by Tukey's post hoc analysis.

Interestingly, some significant proteins had even higher intensity in chitosan + *A. rolfsii* condition than chitosan treatment or *A. rolfsii* infection alone. For example, the signal intensity of thaumatin‐like protein 1 (CS_06) in chitosan treatment (471.4 ± 136.5 arbitrary unit [AU]) was approximately 4.5 times higher than control (104.9 ± 65.2 AU) (Table 1). In chitosan + *A. rolfsii* condition, it was further increased to 1684.3 ± 466.5 AU, approximately 3.6 times higher than that of chitosan treatment. This suggests that after initial elicitation by chitosan, the plant secreted additional defense proteins into the exudate to combat the pathogen. Likewise, the signal intensities of several *A. rolfsii* proteins were significantly increased in chitosan + *A. rolfsii* condition. For example, the signal intensity of exo‐beta‐1,3‐glucanase (AR_01) in *A. rolfsii* inoculation (630.13 ± 277.04 AU) was approximately 5.4 times higher than control (120.99 ± 76.26 AU). It was further increased to 4863.6 ± 440.7 AU in chitosan + *A. rolfsii* condition. This implies that *A. rolfsii* pathogen also increasingly secreted proteins into exudate solution in response to chitosan. It could be assumed that the pathogen might recognize chitosan as additional polysaccharides and produce and secrete more enzymes to digest chitosan molecules.

Surprisingly, secretions of some *C. sativa* proteins were impacted by *A. rolfsii* pathogen but not by chitosan treatment. These proteins are plant enzyme inhibitors, such as Bowman–Birk type proteinase inhibitor 2 (CS_17), proteinase inhibitor (CS_25), and pectinestease inhibitor 44‐like (CS_38). Their protein levels tended to increase in *A. rolfsii* infection and chitosan + *A. rolfsii* conditions. For example, the intensity of Bowman–Birk type proteinase inhibitor 2 (CS_17) was 151.37 ± 141.60 and 53.71 ± 33.80 AU in control and chitosan treatment conditions, respectively. It was increased to 545.50 ± 252.80 and 640.20 ± 149.63 AU in *A. rolfsii* inoculation and chitosan + *A. rolfsii* conditions, respectively (Figure S6 and Table S1). Whereas the secretions of Bowman–Birk type proteinase inhibitor 2 (CS_07) and kunitz trypsin inhibitor 5 (CS_33), the other two proteinase inhibitors identified from this dataset, were likely induced by both chitosan treatment and *A. rolfsii* infection (Figure S6). These results suggest that these enzyme inhibitors are different types of defense proteins and could be triggered via different pathways from the other defense proteins activated by chitosan treatment.

## 4 DISCUSSION

In this study, two interconnected aspects were investigated concomitantly: (1) the pathology of *A. rolfsii* infection on *C. sativa* growth and biochemical defense responses and (2) the priming effect of chitosan to promote plant resistance against the infection. Because the fungal strain acquired from the Biological Collection in Queensland had not been characterized, the fungal species, *A. rolfsii*, was initially confirmed by ITS sequence (Figure S3). Afterwards, the strain was confirmed to cause disease on *C. sativa* by fulfilling Koch's postulates, where the morphology of the fungus isolated from the re‐inoculated plant was identical to the original culture received from the collection (Figure S4). In addition, in vitro fungal growth assays were conducted to validate that .2% chitosan had no fungicidal effect against *A. rolfsii* growth (Figure 1) and was unused by the fungus to support its growth (Figure S5).

After inoculation, *C. sativa* showed signs of southern blight disease, for example, yellowish and wilting leaves, indicating successful infection by the *A. rolfsii* pathogen within 5 days (Figure 2). Total activities of defense enzymes, peroxidase and chitinase, were increased in the shoot tissues, implying plant defense responses were triggered upon the infection (Figure 4). The levels of phytohormones, including IAA (auxin), zeatin (cytokinin), JA, JA‐Ile, and ABA, and metabolites, ICA and CA, were also increased, suggesting that the infection also activated biosynthesis of these hormones and metabolites (Figure 5). Increases of JA hormones were as expected due to their primary roles in plant defense against biotic stress, particularly on necrotrophic pathogens, the pathogen that kills plant hosts and lives off dead cells (Verma et al., 2016; Wang et al., 2021), which is the case for *A. rolfsii*, the pathogen used in this study. Likewise, an increased level of ABA is reasonable due to its interaction with JA signaling pathways, confirming the other studies that suggest synergistic crosstalk between ABA and JA hormones (Ku et al., 2018; Yang et al., 2019). Interestingly, the levels of growth hormones auxin and cytokinin were also increased. Plants might regulate growth hormone productions to rejuvenate new meristems or repair damaged tissues (Akhtar et al., 2019; Bielach et al., 2017). Otherwise, increases in growth hormones might be related to the crosstalk with the SA and JA defense signaling pathways (Shigenaga & Argueso, 2016). ICA is an intermediate molecule in callose deposition process and increase in ICA level was found to induce Arabidopsis resistance against a necrotrophic fungal pathogen, *Plectosphaerella cucumerina* (Gamir et al., 2012; Pastor‐Fernández et al., 2019). ICA also has a similar chemical structure to endogenous auxins and could share the same biosynthesis pathway with auxin hormones (Böttcher et al., 2014). Therefore, changes in auxin productions may affect ICA level. CA is a secondary metabolite, which regulates plant growth and has antioxidant and antimicrobial properties (Singh et al., 2013). The roles of endogenous CA metabolite in plant defense is still unclear, but treating faba bean roots with exogenous CA was shown to reduce plant defense response and increase plant susceptibility to *Fusarium oxysporum* (Zhao et al., 2018). Further research is required to elucidate CA properties in plant biological systems.

Plants primed with chitosan followed by fungal inoculation (chitosan + *A. rolfsii*) still suffered from disease. Shoot morphology and growth parameters of the chitosan‐primed infected plants were not improved from those of the infected plants without priming (Figures 2 and 3). However, the level of defense enzyme, peroxidase, in the shoots of chitosan‐primed infected plants was lower than that of the infected plants (Figure 4). The levels of phytohormones and metabolites including IAA, Me‐IAA, SA, JA, ICA, and CA were also decreased and nearly matched to the control (Figure 5). This implies that root chitosan treatment might level off plant shoot defense responses upon *A. rolfsii* infection. Defense mechanisms might be triggered prior to the inoculation due to chitosan priming, and when plants encountered the pathogen, defense enzyme and hormone levels were not activated to the same levels as observed from the infected only plants. However, chitosan priming might also suppress the production of defense enzymes, phytohormones, and metabolites before the inoculation and lead to the reduced enzyme activity and metabolite levels measured on the final day of observation, because those reductions were also observed in the chitosan treatment condition (Figures 4 and 5). These unknowns lead to a number of hypotheses that can be tested in the future.

Nonetheless, findings from other studies have demonstrated that the effects of chitosan priming and pathogen infection on plant defense enzyme activities and metabolite levels may vary across different timepoints. For example, at 6 days after inoculation, total catalase activity and defense hormone levels of SA and JA were lower in chitosan‐sprayed leaves of apple as compared with the leaves with mock spray upon *Glomerella cingulata* infection (causing leaf spot). The levels of phenolic compounds including catechin, chlorogenic acid, and coumaric acid in chitosan treatment were also lower than those of the infection condition. However, catalase and peroxidase activities and hormone SA level of chitosan‐sprayed plants were comparable or significantly higher than the unsprayed plants at the earlier timepoints at 1–4 days after infection (Liu et al., 2023). In cucumber inoculated with *Erysiphe cichoracearum* (causing powdery mildew), seedlings pre‐soaked with chitosan solution (5 mg mL^−1^) showed lower total polyphenol oxidase and peroxidase activities than the control seedlings at 72 h after infection. Nonetheless, at the earlier timepoints (12–48 h), the enzyme activities measured from chitosan‐primed plants were higher than the unprimed plants (Jogaiah et al., 2020). These data suggest that plant defense responses could be varied during the early to late stage of infection and the eliciting effects of chitosan would not consistently activate plant defense responses over time. Further investigation is required to track down the changes of *C. sativa* defense enzymes and metabolites upon *A. rolfsii* infection in a time‐series pattern.

When compared with shoot tissues, roots were less impaired by *A. rolfsii* infection. Root morphology and growth parameters of the infected plants were not significantly different from the control (Figures 2 and 3). Root defense enzyme activities of the infected plants were also unchanged (Figure 4). The levels of cytokinin and ABA hormone were decreased in the infected plants, but defense hormones, SA and JA, were unaffected (Figure 6). However, in the exudate, proteome profile of *A. rolfsii* infection condition was substantially changed from the control. Cell wall‐degrading enzymes, for example, cellulase, glucanase, amylase, and xylanase were detected in the exudate samples of *A. rolfsii* infection condition but undetected in control and chitosan treatment (Table 1), demonstrating that the pathogen might secrete these digestive enzymes to attack plant root systems. In chitosan + *A. rolfsii* condition, the enzyme levels were even higher than *A. rolfsii* infection alone, suggesting that the existence of chitosan in the root‐growth chamber of the Root‐TRAPR system might induce *A. rolfsii* pathogen to increase enzyme secretions. It is possible that *A. rolfsii* might recognize chitosan as an additional glycosidic molecule, in addition to polysaccharides in plant cell walls, and secreted more glycoside hydrolase enzymes to digest chitosan polymer. However, the pathogen does not appear to intentionally break down chitosan to support its growth as increasing growth of the fungus was not observed from the chitosan condition in the in vitro assay using minimal YNB media (Figure S5).

Interestingly, a few *C. sativa* secreted proteins were affected by *A. rolfsii* infection. These proteins were not initially elicited by chitosan but were specifically secreted upon *A. rolfsii* pathogen infection (Figures 9 and S6). They were enzyme inhibitors, such as Bowman–Birk type proteinase inhibitor 2 and pectinesterase inhibitor. Their secretions might occur after the plant recognized the presence of specific fungal proteins. For instance, pectinesterase inhibitor 44‐like (CS_38) might be secreted once the plant detected fungal pectinesterase enzymes (AR_02). Likewise, the plant might secrete proteinase inhibitors (CS_17 and CS_25) when encountering fungal serine proteinases, such as trypsin and chymotrypsin according to their functions (Casaretto & Corcuera, 1995). However, these fungal proteinase enzymes were not identified from our proteome dataset likely because bovine trypsin, used for protein cleavage in proteomics analysis, was considered as a contaminant. Based on BLAST results, bovine trypsin (UniProt ID: P00760) and *F. oxysporum* trypsin (UniProt ID: P35049), the only well‐annotated fungal trypsin in the database, share approximately 40% sequence homology. Therefore, it is possible that some fungal tryptic peptides were filtered out as contaminants during data processing steps and lost from the protein identification list. Alternative use of other digestive enzymes such as LysC, GluC, and ArgC for protein digestion may instead enable the detection of fungal trypsin enzymes (Giansanti et al., 2016). Plant proteinase inhibitors are also classified as PR proteins (PR‐6) but in different families from other defense proteins, i.e. PR‐1 proteins, thaumatin‐like proteins (PR‐5), peroxidases (PR‐9), and chitinases (PR‐3, PR‐8, and PR‐11) (Ferreira et al., 2007). Our results suggest that the roles of enzyme inhibitors in plant defense might be different from other defense proteins, and their expressions might be triggered via different channels. Further investigation is required to resolve specific roles and activation pathways of these plant enzyme inhibitors upon fungal pathogenesis.

In contrast, chitosan significantly altered *C. sativa* root physiology and biochemical responses regardless of pathogen infection. Plant defenses were promoted, where total chitinase activity was increased in the root tissue and total peroxidase activity was increased in the root exudate (Figure 4). Plant defense proteins such as PR proteins, thaumatin‐like proteins, peroxidases, and chitinases were identified from the exudate of chitosan‐treated plants but scarcely found in the exudates from control and *A. rolfsii*‐infected plants (Figure 9 and Table 1). Interestingly, some proteins, such as thaumatin‐like protein 1 and superoxide dismutase, had significantly higher intensities in chitosan + *A. rolfsii* exudates, suggesting that the plant might additionally secrete these defense proteins after encountering the pathogen (Table S1). Nevertheless, chitosan treatment has a root‐growth inhibitory effect, where root length and surface area of chitosan‐treated plants were significantly smaller than those of the untreated plants (Figures 2 and 3a,b). These findings confirm the chitosan effects on *C. sativa* root systems in buttressing plant defense but forfeiting root expansion (Suwanchaikasem et al., 2023). The similar outcome of chitosan inhibiting root growth but promoting plant defense was also found in Arabidopsis studies (Iglesias et al., 2019; Lopez‐Moya et al., 2017). This compromising process is known as the “plant growth‐defense tradeoff,” commonly triggered by biotic and abiotic stresses, including light, water, nutrients, insects, pests, and microorganisms (He et al., 2022). Our findings reveal that chitosan could be another factor driving this shift.

To date, molecular mechanisms underlying the plant growth‐defense tradeoff have not been clearly understood. Several reports suggest that phytohormones and their signaling pathways could be leading compounds and backbone circuits, modulating this balance (Cunha da Silva et al., 2019; He et al., 2022; Huot et al., 2014). In this study, root JA and its derivatives, JA‐Ile and OPDA, were significantly increased upon chitosan treatment. JA signaling pathway is a plant defense pathway against necrotrophic pathogens (Li et al., 2022). It also interacts with other hormonal pathways to regulate plant growth and other stress responses (Li et al., 2022). Therefore, JA and its derivatives, JA‐Ile and OPDA, levels and their signaling pathways could be one of the players involved in chitosan‐induced plant defense promotion. Root CA level was also significantly increased upon chitosan treatment. Exogenous CA was found to influence root auxin biosynthesis and efflux, leading to an inhibition of primary root growth but promotion of lateral root formation (Steenackers et al., 2017). Hence, CA metabolite could be one of the factors contributing to root‐growth hindrance in this study. To verify and confirm the roles of these compounds on root growth‐defense tradeoff, further studies could implement basic functional analyses, for example, challenging plant roots with hormone inhibitors or supplementing exogenous hormones into growth media and monitoring plant root responses. Otherwise, molecular techniques such as virus‐induced gene silencing (VIGS) could be conducted.

In terms of disease control, chitosan treatment in the hydroponic solution was not observed to protect *C. sativa* against *A. rolfsii* infection. This could be because, in this experiment, the *A. rolfsii* pathogen was found to impact mainly the plant shoots, although in field conditions, disease symptoms are detected from both plant shoot and root systems (Joy & Hudelson, 2019; Pfeufer et al., 2018). We hypothesize that the protective effect of chitosan would be more pronounced if the experiment was conducted with other pathogens, such as those that specifically colonize and infect root tissues. One example is the oomycetes, a water‐borne pathogen whose zoospores survive in aqueous solution and can encyst plant roots (Hardham, 2007; Kamoun et al., 2015). To further examine the potential of root chitosan treatment in hydroponic solution to protect plants from *A. rolfsii* fungal attack, the concentration of fungal inoculum could be adjusted to doses that do not kill the plants quickly, so that plant responses can be observed in different degrees. Moreover, further investigation could apply chitosan on shoot tissues, for example, by foliar spraying to direct the treatment to the site of infection. Optimizing chitosan concentrations, timing, duration, and frequency of usage would be another domain to explore to maximize the effects of chitosan treatment. Chitosan has also been used in combination with other bioagents such as beneficial bacteria, fungi, or seaweed extract. The synergistic results of combining treatments to induce plant resistance against southern blight disease have been convincingly demonstrated (Ahmed et al., 2019; de Souza et al., 2018; Gunupuru et al., 2019). Transforming normal‐sized chitosan into nanoparticles would be another method to enhance eliciting properties of chitosan (Chun & Chandrasekaran, 2019; Siddaiah et al., 2018). Although a number of studies have revealed the promising capability of chitosan and its derivatives to combat fungal diseases, good farming husbandries such as avoiding fields with disease history, zero or simplified tillage farming, and rotation with non‐host crops are still fundamental practices to enhance the success rate of disease management in a sustainable manner (Berlin et al., 2018; Różewicz et al., 2021).

## 5 CONCLUSION

In a hydroponic system, *A. rolfsii* pathogen infected *C. sativa*, highly affecting shoot growth and causing yellowish, drooping leaves. Upon infection, plant shoot defense systems were activated, evidenced by increased defense hormones and enzymatic activities. Root chitosan priming failed to prevent disease progression, but interestingly plant defense responses, including activity of defense enzymes and hormone levels, were not as high as observed in the unprimed infected plants. Pathogen‐secreted enzymes, including pectinesterase, xylanase, glucanase, and amylase, were observed in the hydroponic solution, but no evidence of root infection was detected. Chitosan priming strongly promoted root defense but suppressed root growth regardless of fungal infection. This finding confirms the effect of chitosan to regulate root a growth‐defense tradeoff. JA hormone and its derivatives, JA‐Ile and OPDA, may be functional molecules, corresponding to the switch from expanding root growth to prioritizing defense. The results suggest that chitosan has potential to enhance *C. sativa* plant defense against fungal diseases like southern blight, but further research is required to optimize chitosan dosage, formulation, and method of application to maximize chitosan efficacy and promote its utilization.

## AUTHOR CONTRIBUTIONS

Pipob Suwanchaikasem, Alexander Idnurm, Jamie Selby‐Pham, Robert Walker, and Berin A. Boughton designed the study. Pipob Suwanchaikasem conducted the experiments and analyzed the data. Shuai Nie performed proteomics analysis. Alexander Idnurm, Jamie Selby‐Pham, Robert Walker, and Berin A. Boughton provided guidance and technical support throughout the study. Pipob Suwanchaikasem prepared the manuscript. All authors edited and approved the final version.

## ACCESSION NUMBERS

*A. rolfsii* ITS gene sequences: GenBank OP369074 and OP369075.

## CONFLICT OF INTEREST STATEMENT

This work was partly financially supported by Nutrifield Pty Ltd, but the findings of this study are not used for any commercial purpose.

## Supporting information


**Figure S1** Simplified fungal inoculation procedure. A 5‐mm mycelial disc of *Athelia rolfsii* was excised from a PDA plate with full mycelia growth and introduced to *Cannabis sativa* , adjacent to the plant crown and floating on the plant‐solution interface in the Root‐TRAPR system as shown in white circle. The zoomed‐in circle indicates exact position of the mycelial disc in the system as pointed by white arrow.

**Figure S2** Testing pathogenesis of four fungal pathogens isolated from diseased *Cannabis sativa* including *Athelia rolfsii*, *Macrophomina phaseolina*, *Fusarium* sp. and *Phomopsis* sp. A 5‐mm mycelial disc of each fungal pathogen was introduced to 4–6‐days old *C. sativa* seedlings grown in the Root‐TRAPR system using the same method as shown in Figure S1. Five days later, the disease progression was monitored. Three biological replicates were performed per condition. Representative images were depicted for presentation.

**Figure S3** Phylogenetic tree of *Athelia rolfsii* ITS gene sequences. Two copies derived from the strain BRIP 39302a (accessions OP369074 and OP369075) were compared to the other *A. rolfsii* sequences retrieved from the NCBI database. Plant host and country of origin of each isolate are stated after fungal species name. Bootstrap value (%) from 1000 replications is shown within the tree and the sequence from *Sclerotium hydrophilum* was used as an outgroup.

**Figure S4** Confirmation of the organism causing southern blight disease. *Athelia rolfsii* strain BRIP 39302a was isolated off diseased *Cannabis sativa* in Queensland in 2003. It was identified and stored in the Biological Collections, Department of Agriculture and Fisheries, Queensland before shipped to Victoria for this study. In the experiment, seedlings were transferred to the Root‐TRAPR system on *Day 0* and primed with chitosan on *Day 6*. On the inoculation day (*Day 8*), *A. rolfsii* pathogen was introduced to the plants in two conditions, *A. rolfsii* inoculation and chitosan + *A. rolfsii* conditions. Five days after inoculation *(Day 13)*, small parts of stem and root were excised and cultured in the PDA plate supplied with antibiotics. After seven days of incubation, the pattern of fungal growth was examined. Representative images of *A. rolfsii* infection and control are shown. Eight replicates were performed for control and chitosan conditions and twelve replicates were performed for *A. rolfsii* inoculation and chitosan + *A. rolfsii* conditions. Representative images of *A. rolfsii* infection and control were depicted for presentation. This figure was partly created using Biorender.com and Freepik.com.

**Figure S5** Images of *Athelia rolfsii* growth in the yeast nitrogen base (YNB) media, containing 0.2% and 0.5% chitosan or glucose. White particles observed in chitosan conditions were insoluble parts of chitosan after dispersion in YNB media. Three biological replicates were performed per condition. Representative images were depicted for presentation.

**Figure S6** Boxplot intensity of five proteinase inhibitors identified from this exudate proteome dataset; CS_07: Bowman‐Birk type proteinase inhibitor 2, CS_17: Bowman‐Birk type proteinase inhibitor 2, CS_25: Proteinase inhibitor, CS_33: kunitz trypsin inhibitor 5 and CS_38: pectinesterase inhibitor 44‐like. The boxplots display interquartile range box, whiskers, median and outliers. Letters (A‐B) refer to statistically significant difference (*q* < 0.05) using one‐way ANOVA with permutation‐based FDR, followed by Tukey's post hoc analysis. NS refers to a non‐significant difference (*q* ≥ 0.05) across four sample groups. Full protein identification and statistical data are supplied in Table S1.

**Table S1.** Full protein identification and statistical details of exudate proteins

**Table S2.** Raw measurements and statistical analysis of plant growth parameters, enzymatic activities and phytohormone quantification

## Data Availability

The data that support the findings of this study are available within the paper or in the Supporting Information.
