# Supplementary figures and images for: Hormonal and proteomic analyses of southern blight disease caused by Athelia rolfsii and root chitosan priming on Cannabis sativa in an in vitro hydroponic system
Source: Plant Direct. 2023 Sep 8;7(9):e528. doi: 10.1002/pld3.528 (PMC10485662; doi:10.1002/pld3.528)

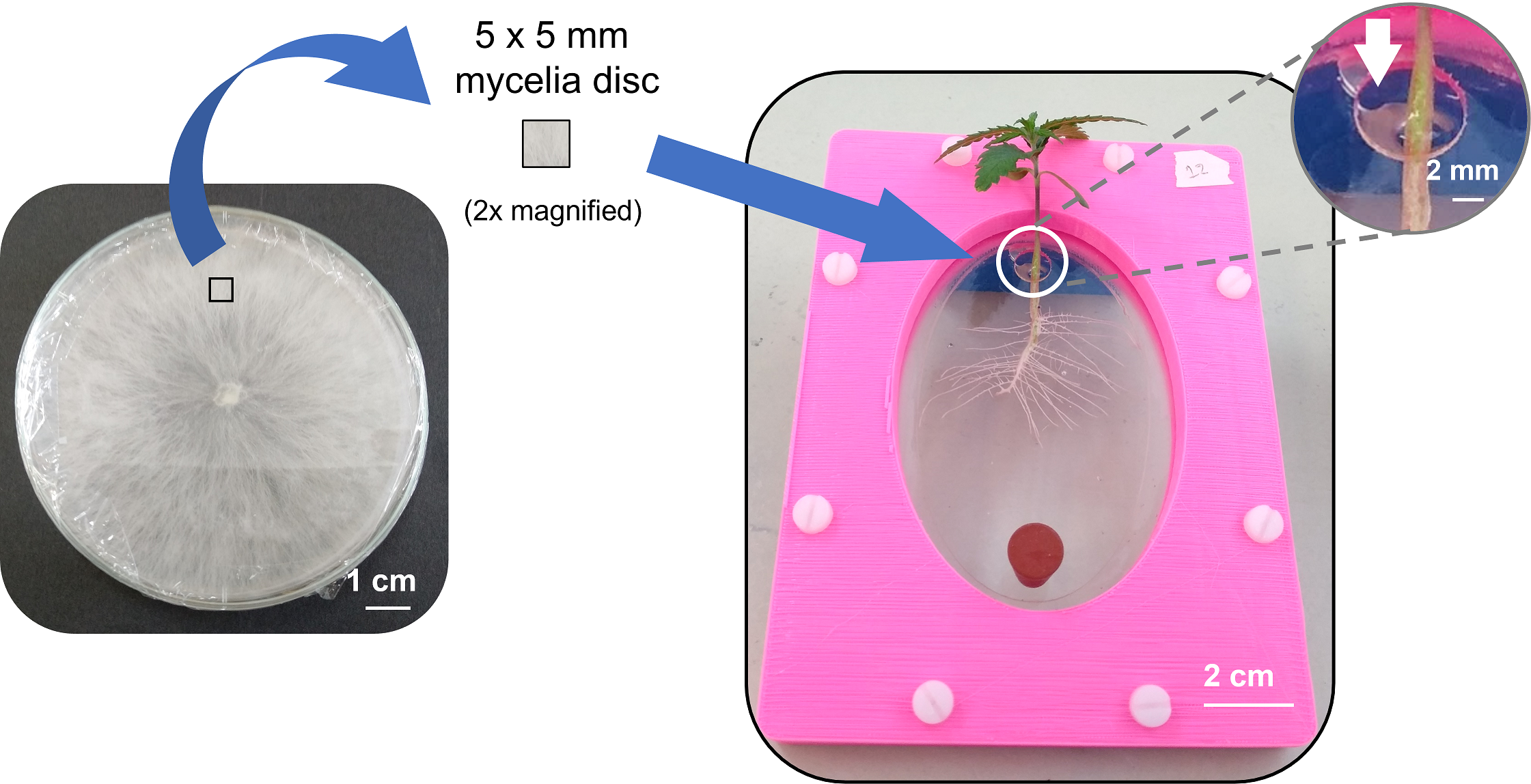

Supplement: Supplementary file 1 — Figure S1 Simplified fungal inoculation procedure. A 5‐mm mycelial disc of Athelia rolfsii was excised from a PDA plate with full mycelia growth and introduced to Cannabis sativa , adjacent to the plant crown and floating on the plant‐solution interface in the Root‐TRAPR system as shown in white circle. The zoomed‐in circle indicates exact position of the mycelial disc in the system as pointed by white arrow. [file PLD3-7-e528-s004.tif]

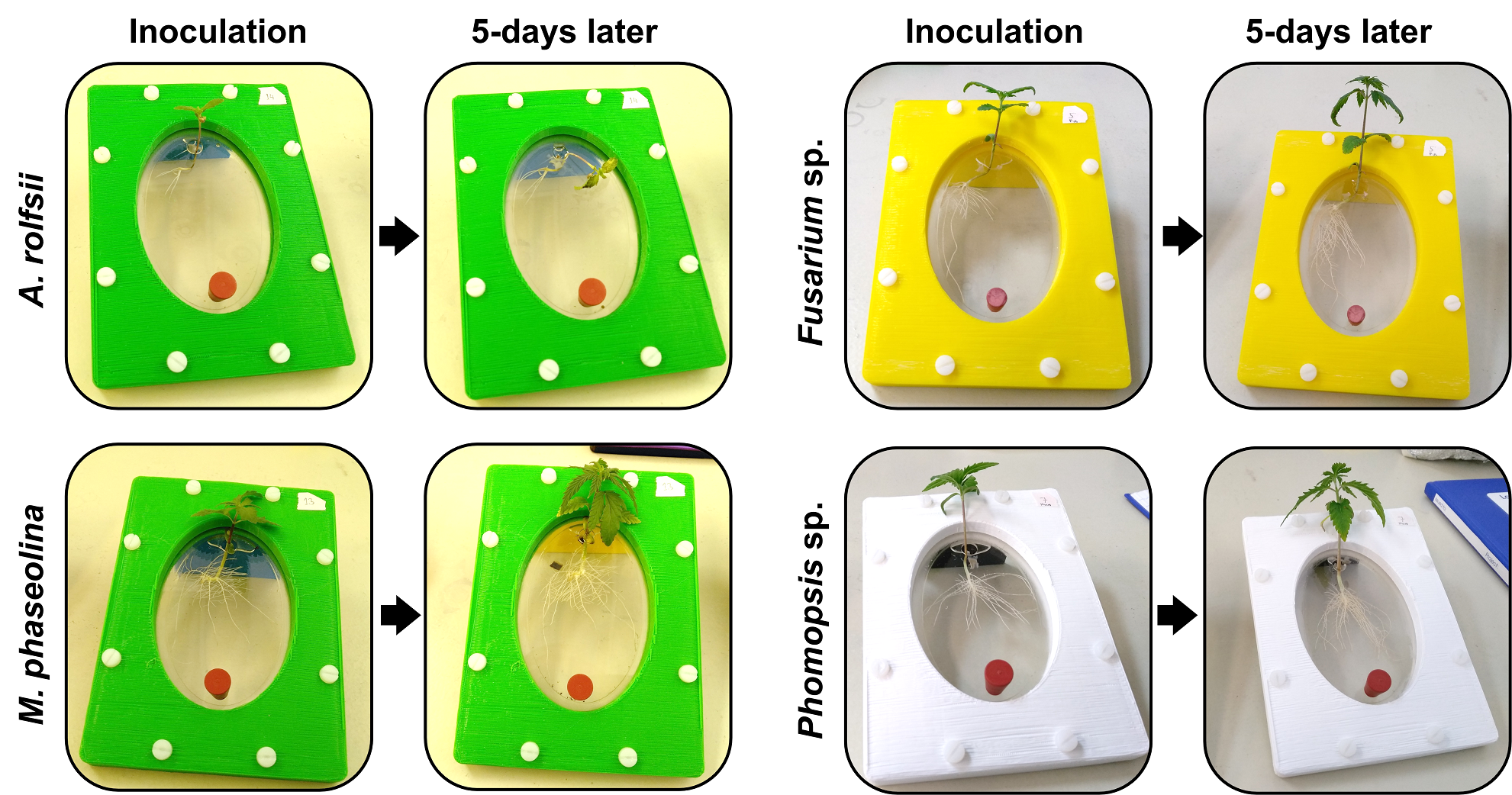

Supplement: Supplementary file 2 — Figure S2 Testing pathogenesis of four fungal pathogens isolated from diseased Cannabis sativa including Athelia rolfsii, Macrophomina phaseolina, Fusarium sp. and Phomopsis sp. A 5‐mm mycelial disc of each fungal pathogen was introduced to 4–6‐days old C. sativa seedlings grown in the Root‐TRAPR system using the same method as shown in Figure S1. Five days later, the disease progression was monitored. Three biological replicates were performed per condition. Representative images were depicted for presentation. [file PLD3-7-e528-s006.tif]

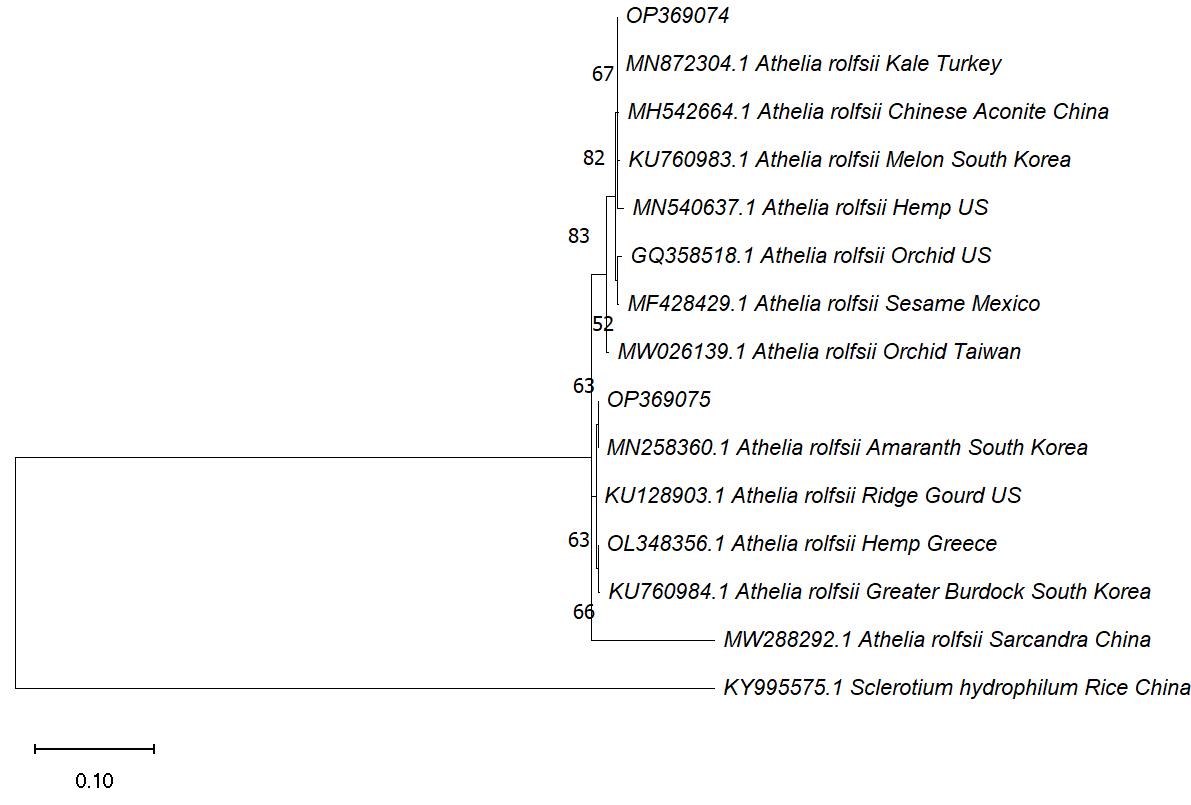

Supplement: Supplementary file 3 — Figure S3 Phylogenetic tree of Athelia rolfsii ITS gene sequences. Two copies derived from the strain BRIP 39302a (accessions OP369074 and OP369075) were compared to the other A. rolfsii sequences retrieved from the NCBI database. Plant host and country of origin of each isolate are stated after fungal species name. Bootstrap value (%) from 1000 replications is shown within the tree and the sequence from Sclerotium hydrophilum was used as an outgroup. [file PLD3-7-e528-s003.tif]

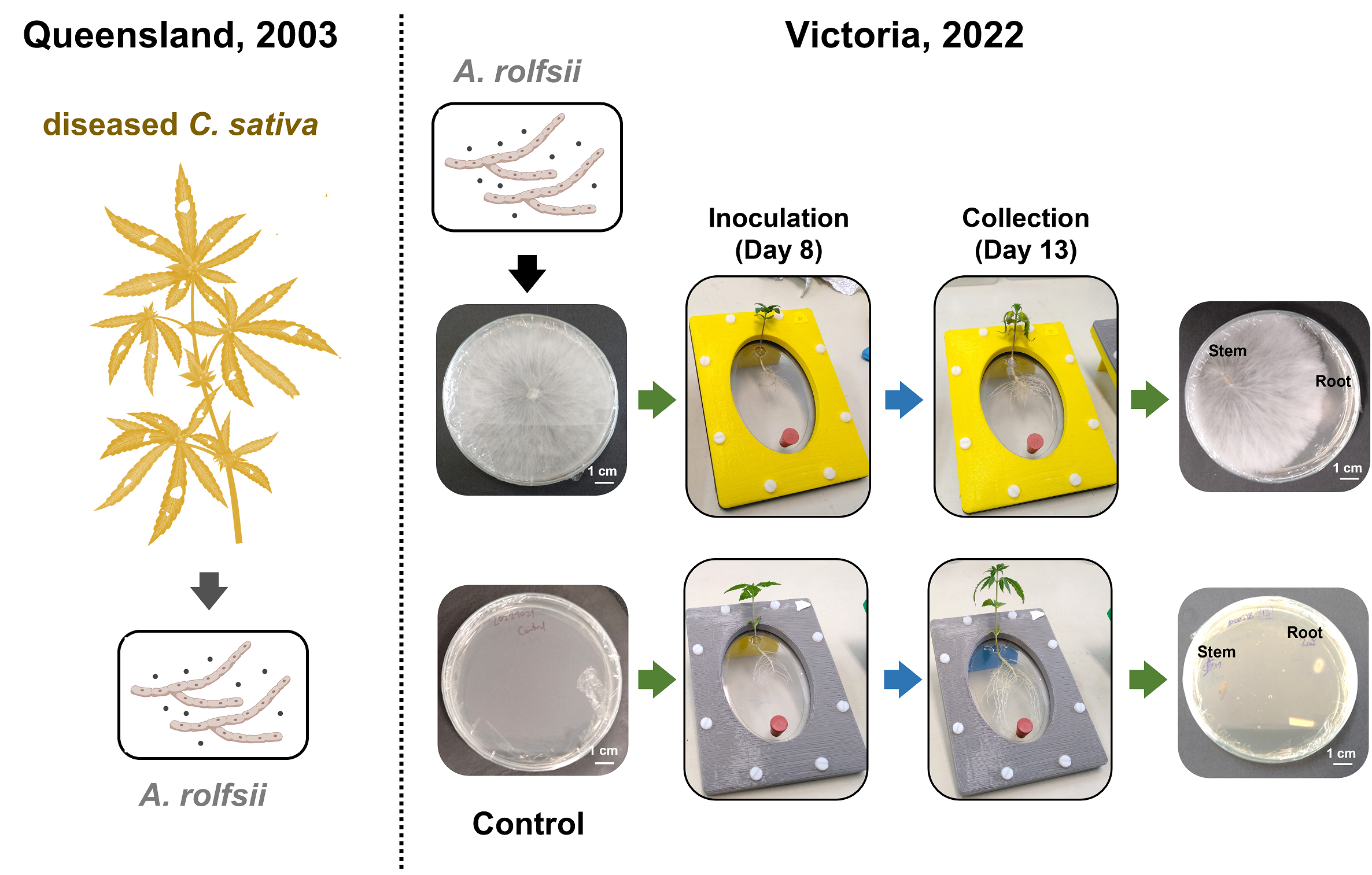

Supplement: Supplementary file 4 — Figure S4 Confirmation of the organism causing southern blight disease. Athelia rolfsii strain BRIP 39302a was isolated off diseased Cannabis sativa in Queensland in 2003. It was identified and stored in the Biological Collections, Department of Agriculture and Fisheries, Queensland before shipped to Victoria for this study. In the experiment, seedlings were transferred to the Root‐TRAPR system on Day 0 and primed with chitosan on Day 6. On the inoculation day (Day 8), A. rolfsii pathogen was introduced to the plants in two conditions, A. rolfsii inoculation and chitosan + A. rolfsii conditions. Five days after inoculation (Day 13), small parts of stem and root were excised and cultured in the PDA plate supplied with antibiotics. After seven days of incubation, the pattern of fungal growth was examined. Representative images of A. rolfsii infection and control are shown. Eight replicates were performed for control and chitosan conditions and twelve replicates were performed for A. rolfsii inoculation and chitosan + A. rolfsii conditions. Representative images of A. rolfsii infection and control were depicted for presentation. This figure was partly created using Biorender.com and Freepik.com. [file PLD3-7-e528-s007.tif]

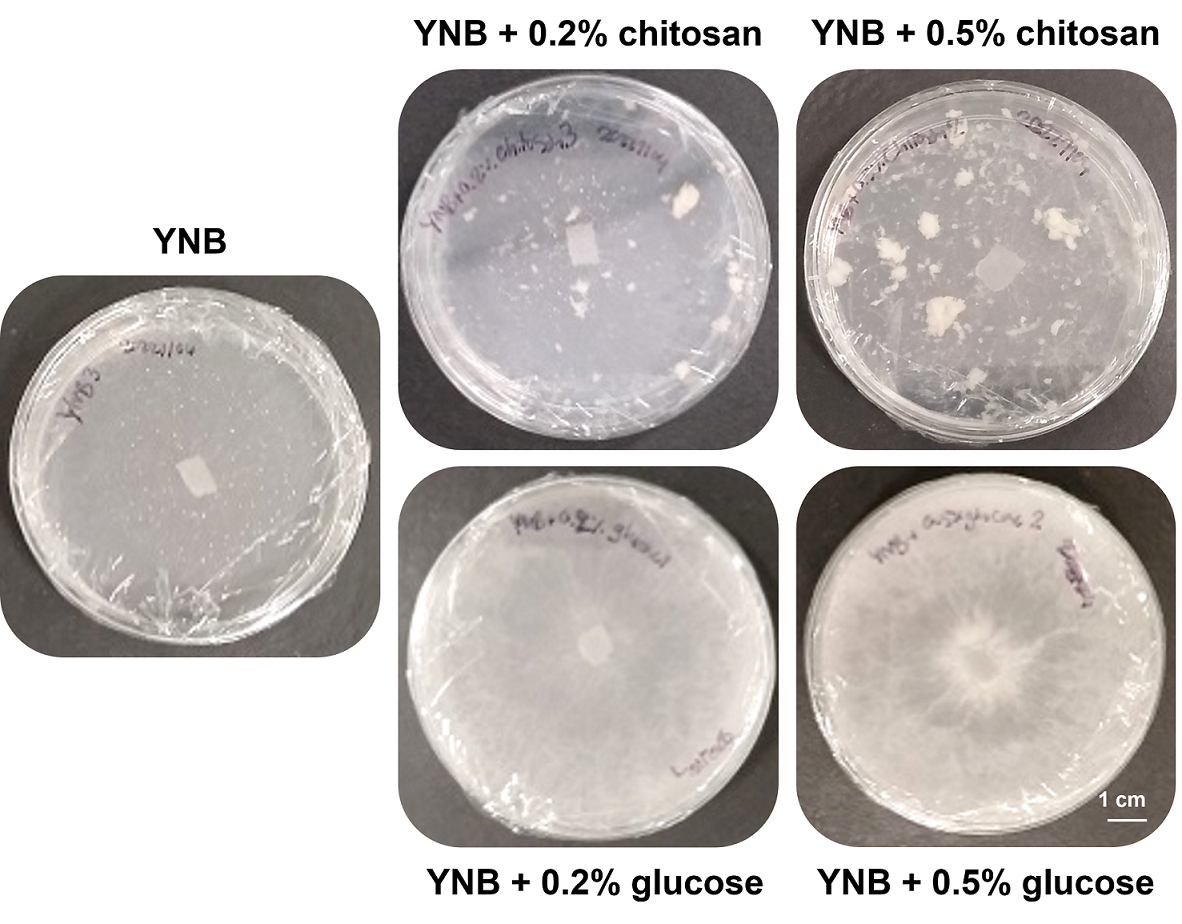

Supplement: Supplementary file 5 — Figure S5 Images of Athelia rolfsii growth in the yeast nitrogen base (YNB) media, containing 0.2% and 0.5% chitosan or glucose. White particles observed in chitosan conditions were insoluble parts of chitosan after dispersion in YNB media. Three biological replicates were performed per condition. Representative images were depicted for presentation. [file PLD3-7-e528-s005.tif]

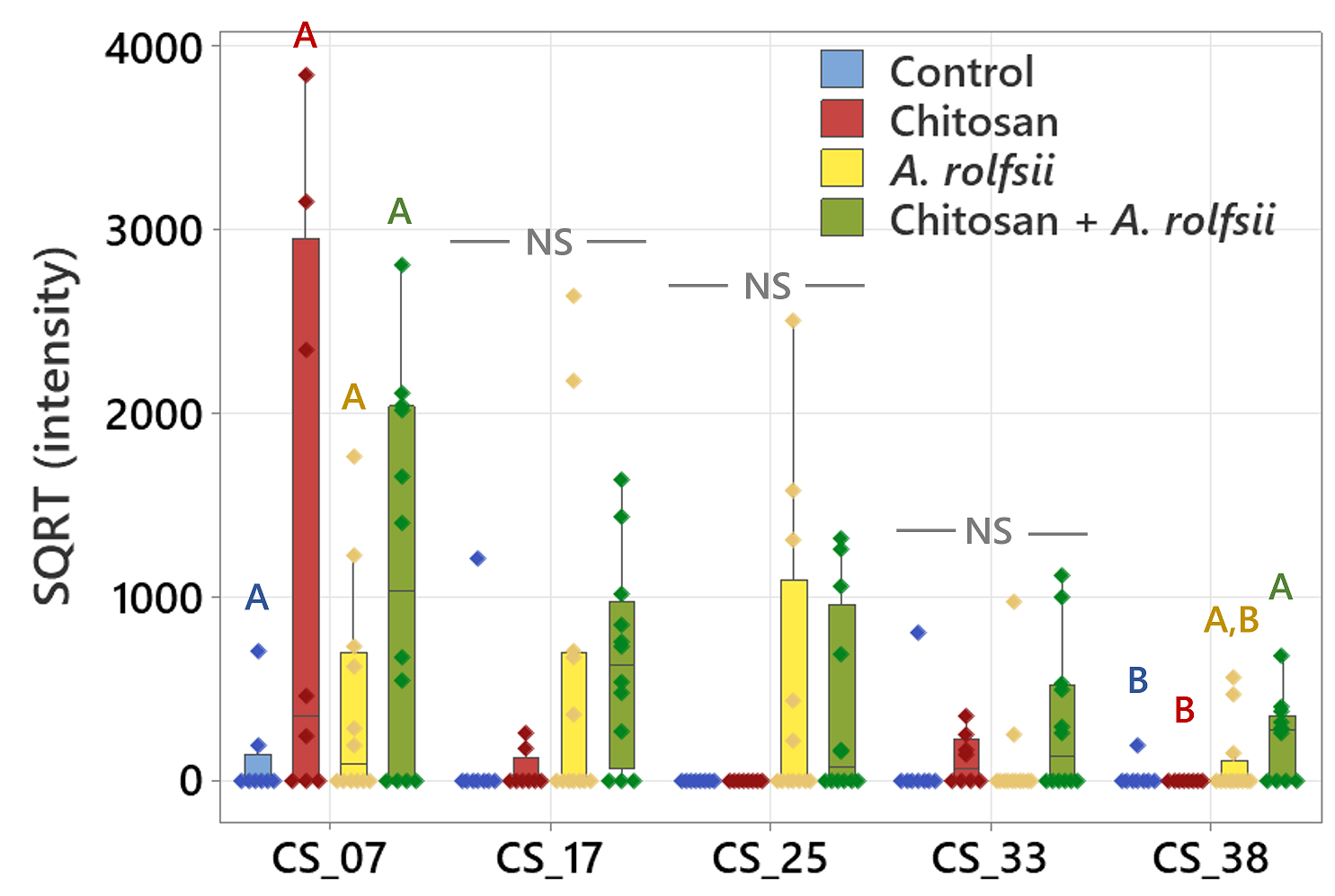

Supplement: Supplementary file 6 — Figure S6 Boxplot intensity of five proteinase inhibitors identified from this exudate proteome dataset; CS_07: Bowman‐Birk type proteinase inhibitor 2, CS_17: Bowman‐Birk type proteinase inhibitor 2, CS_25: Proteinase inhibitor, CS_33: kunitz trypsin inhibitor 5 and CS_38: pectinesterase inhibitor 44‐like. The boxplots display interquartile range box, whiskers, median and outliers. Letters (A‐B) refer to statistically significant difference (q < 0.05) using one‐way ANOVA with permutation‐based FDR, followed by Tukey's post hoc analysis. NS refers to a non‐significant difference (q ≥ 0.05) across four sample groups. Full protein identification and statistical data are supplied in Table S1. [file PLD3-7-e528-s002.tif]
